# Network modeling of patients' biomolecular profiles for clinical phenotype/outcome prediction

**DOI:** 10.1038/s41598-020-60235-8

**Published:** 2020-02-27

**Authors:** Jessica Gliozzo, Paolo Perlasca, Marco Mesiti, Elena Casiraghi, Viviana Vallacchi, Elisabetta Vergani, Marco Frasca, Giuliano Grossi, Alessandro Petrini, Matteo Re, Alberto Paccanaro, Giorgio Valentini

**Affiliations:** 1 AnacletoLab - Dipartimento di Informatica, Università degli Studi di Milano, Milan, 20133 Italy; 2 Department of Dermatology, Fondazione IRCCS Ca’ Granda - Ospedale Maggiore Policlinico, Milan, 20122 Italy; 3 Unit of Immunotherapy of Human Tumors, Fondazione Istituto di Ricovero e Cura a Carattere Scientifico (IRCCS) Istituto Nazionale dei Tumori di Milano, Milan, Italy; 4 Royal Holloway, University of London, Centre for Systems and Synthetic Biology - Department of Computer Science, Egham, TW20 0EX UK; 5 School of Applied Mathematics, Fundação Getulio Vargas, Rio de Janeiro, Brazil

**Keywords:** Molecular medicine, Computer science

## Abstract

Methods for phenotype and outcome prediction are largely based on inductive supervised models that use selected biomarkers to make predictions, without explicitly considering the functional relationships between individuals. We introduce a novel network-based approach named *Patient-Net (P-Net)* in which biomolecular profiles of patients are modeled in a graph-structured space that represents gene expression relationships between patients. Then a kernel-based semi-supervised transductive algorithm is applied to the graph to explore the overall topology of the graph and to predict the phenotype/clinical outcome of patients. Experimental tests involving several publicly available datasets of patients afflicted with pancreatic, breast, colon and colorectal cancer show that our proposed method is competitive with state-of-the-art supervised and semi-supervised predictive systems. Importantly, *P-Net* also provides interpretable models that can be easily visualized to gain clues about the relationships between patients, and to formulate hypotheses about their stratification.

## Introduction

Phenotype and outcome prediction using sets of selected biomarkers are well-established prediction tasks in the context of computational biology, including different prediction problems ranging from the response to a specific drug^1,2^, diagnosis and prognosis^3–5^, classification of cancer subtypes^6^, outcome and recurrence prediction^7–9^ and other related prediction problems^10^. State-of-the-art methods for these problems are largely based on inductive supervised models that use sets of selected biomarkers, usually represented as vectors, to predict the phenotype or outcome of interest (see, e.g.^11–13^), without taking into account the relationships between individuals.

Several works proposed “network-based” methods by constructing graphs of patients, in order to discover the underlying structure of the data (e.g. discovery of subtypes of diseases, clinical stratification of patients)^14–17^. These methods mainly used unsupervised approaches and hence have been not specifically designed and are not appropriate for phenotype/outcome prediction problems. Recently a few works proposed semi-supervised “network-based” approaches for the prediction of the phenotype/outcome of patients, on the basis of their bio-molecular profiles (e.g. gene expression of genotypic profiles)^18,19^, including also methods able to integrate multiple sources of omics data^20^, and methods based on Supervised Random Walks^21^, specifically modified for the classification of tumors^22^.

In this work, we introduce a novel network-based method for modeling in the “patient space”. In this context the nodes of the network represent patients through an *n*-dimensional set of biomarker values (e.g. a set of gene expression values), and edges represent similarities between the biomarkers of a pair of patients. Hence, this “patient-space” differs from the classical “biomarker-space”, where nodes represent biomarkers and edges similarities between biomarkers and not between patients^23,24^. More precisely, we construct networks of patients on the basis of their gene expression similarities (e.g. by considering their expression profiles), and then we apply a semi-supervised transductive method to predict their phenotype or clinical outcome. The algorithm leverages local learning strategies, by considering the direct neighbors of each node in the “patient network”, as well as global topological characteristics of the net through the adoption of appropriate graph-kernels^25^.

Our method, that we named *P-Net* (i.e. Patient-Net), uses the available a priori knowledge about the phenotype/outcome of patients and their biomolecular similarity, to assign a score and to rank or to classify patients according to the phenotype/outcome under study.

Importantly, P-Net, differently from classical inductive supervised models, is not a mere “black box”, since the visual inspection of the network can unravel further characteristics of the patients under study. Indeed, by exploiting a cytoscape.js interface, and some rendering options available in the package, it is possible to explore and graphically analyze the obtained graph. It is worth noting that P-Net is also completely different from semi-supervised network-based methods where genes or proteins represent the main object to be studied^24^. In fact these methods, by exploiting the topological relationships between nodes in gene and protein networks, can select markers for specific diseases^5,26–32^, but cannot be directly applied to predict the outcome or phenotype of patients, which is instead the main aim of *P-Net*.

A large set of experiments with real biomolecular data, including Pancreatic, Breast, Colorectal and Colon cancer patients, shows that *P-Net* is competitive with supervised and semi-supervised state-of-the-art methods for phenotype/outcome prediction. A fast and efficient implementation of *P-Net* is publicly available from GitHub (https://github.com/GliozzoJ/P-Net). Moreover, by using a cytoscape.js interface, our method offers an intuitive way to explore and graphically analyze the patient network.

## Methods

### The *P-Net* algorithm

Network-based ranking of patients with respect to a given phenotype (*P-Net*) is a semi-supervised algorithm able to assign to each patient a score related to its odds to show a specific phenotype (e.g. clinical outcome, response to treatment). The predictor is constructed from patients’ molecular profiles using a graph *G* = <*V*, *E*>, where the set of vertices *V* corresponds to patients and the set of edges *E* to relationships between them (e.g. correlation of expression profiles or correlation of clinical features associated with each patient). From this similarity network among patients, a graph kernel (e.g. a random walk kernel) is applied to obtain weighted edges aware of the global topology of the network^33,34^, while edges with low weight are removed through a cross-validation procedure. Finally, a scoring system, by exploiting a subset *V*_*C*_ ⊂ *V* of labelled patients belonging to the subgroup *C* of interest (e.g. patients having poor prognosis or responsive to a specific treatment), assigns a score to each patient on the basis of the labeling of its neighborhood. The resulting ranking of patients can be used to rank or classify them by their likelihood to show a specific *C* phenotype.

 Figure 1 summarizes the main logical steps of *P-Net*:

1. **Data collection and feature selection**. The biomolecular profiles of *n* patients are collected as columns in a matrix ***M***. Its *m* rows refer to the features, e.g. DNA microarray or RNA-seq gene expression measurements, or epigenetic data (Step 1 of Fig. 1). In other words, the columns of ***M*** represent the biomolecular profiles of patients, while the rows the genomic features associated with each patient. Since usually *m* largely outnumbers *n*, we apply a feature selection method to reduce the dimensionality and select the most relevant features for the phenotype/outcome under study. In our experiments, we used the t-test to select the first ranked $m{\prime}  < m$ features available in the training set.

**Figure 1:**
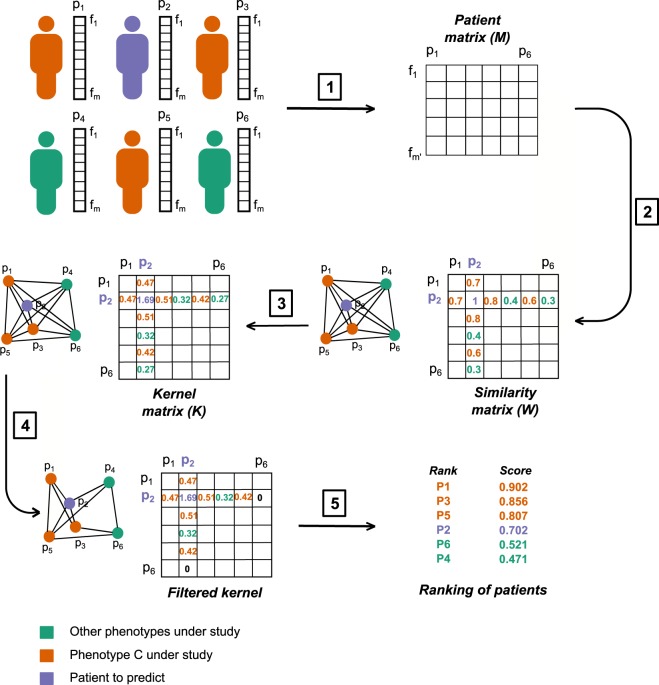
Main logical steps of the P-Net algorithm. For simplicity the graph is shown with *n* = 6 patients. (1) Each patient is represented by a vector of *m* features (e.g. expression levels of the genes). Orange patients have the *C* phenotype; green patients do not show the *C* phenotype; violet patients are not labelled and our goal is to predict their label. We select the $m{\prime}  < m$ features most correlated with the phenotype *C* and we use them to construct a $m{\prime} \times n$ matrix whose *n* columns represent the bio-molecular profiles of patients restricted to the ${m}^{{\prime} }$ selected features. (2) A *W* similarity matrix is constructed, where each element *w*_*ij*_ represents, e.g., the positive filtered Pearson correlation between the patients *i* and *j*; note that this matrix can be interpreted as the adjacency matrix of the graph of patients. To make the figure readable we show only the entries of patient *p*_2_. (3) The corresponding *K* kernel matrix is computed through e.g. 2-step Random Walk Kernel. (4) The *K* matrix is filtered and we remove all the edges with a weight lower than the selected threshold *τ* = 0.32. (5) A *score function* (e.g. Nearest Neighbour score) is finally used to compute a score for each patient and to rank them according to their scores.
2. **Construction of the patient similarity matrix** A matrix ***W*** of similarities between biomolecular profiles (full biomolecular profiles or signatures) is obtained from the columns (patients) of ***M*** (step 2 of Fig. 1). To construct ***W*** we used the filtered Pearson correlation (by setting to zero all negative values), but other measures (e.g. the Spearman correlation or the inverse of some distances, such as the Euclidean or the Manhattan distance) can be used instead. We applied filtered Pearson correlation because other correlation metrics achieved similar or worse results. ***W*** can be seen as the adjacency matrix of the weighted graph *G*, where the edge weights represent the bio-molecular similarity between patients.
3. **Computation of a kernel matrix from the patient similarity matrix** The kernel matrix ***K*** is derived from the similarity matrix ***W*** (Fig. 1 - Step 3). This is achieved by choosing a *kernel*^35^, able to capture the topological characteristics of the underlying graph. Intuitively, the idea is to derive a new graph where there is an edge between each pair of nodes that is highly connected (i.e. there exist many short paths between them), in the original graph. This means that two nodes may be directly connected in the new graph even if they were not in the original graph (see Fig. 2). Several kernels are suitable for this problem (e.g. the diffusion kernel^36^), but for our experiments we chose the random walk kernel^37,38^, since it showed on average better results:
  $${\boldsymbol{K}}=(a-1){\boldsymbol{I}}+{{\boldsymbol{D}}}^{-\frac{1}{2}}{\boldsymbol{W}}{{\bf{D}}}^{-\frac{1}{2}}$$
  where ***I*** is the identity matrix, ***D*** is the “degree” diagonal matrix with elements *d*_*ii*_ = ∑_*j*_*w*_*ij*_ and *a* is a value larger than 2. A *p-step* random walk kernel can be obtained by simply multiplying ***K*** by itself *p* times. Intuitively, we can think at graph-kernels as functions able to enrich the similarity between nodes in the transformed kernel space, since the novel edge weights of ***K*** is determined by the overall topology of the network (e.g. novel edges between vertices are added if a path between them of length equal or less than the steps of the random walk does exist)^39^. It is worth noting that a graph kernel implicitly induces a new non linear similarity measure between patients, since the novel weights of the computed Gram matrix (i.e. the similarity patient matrix resulting from the application of the kernel) take into account both the topology and the metric distance between the nodes/patients to construct a novel “enriched” similarity graph (Fig. 2).

**Figure 2:**
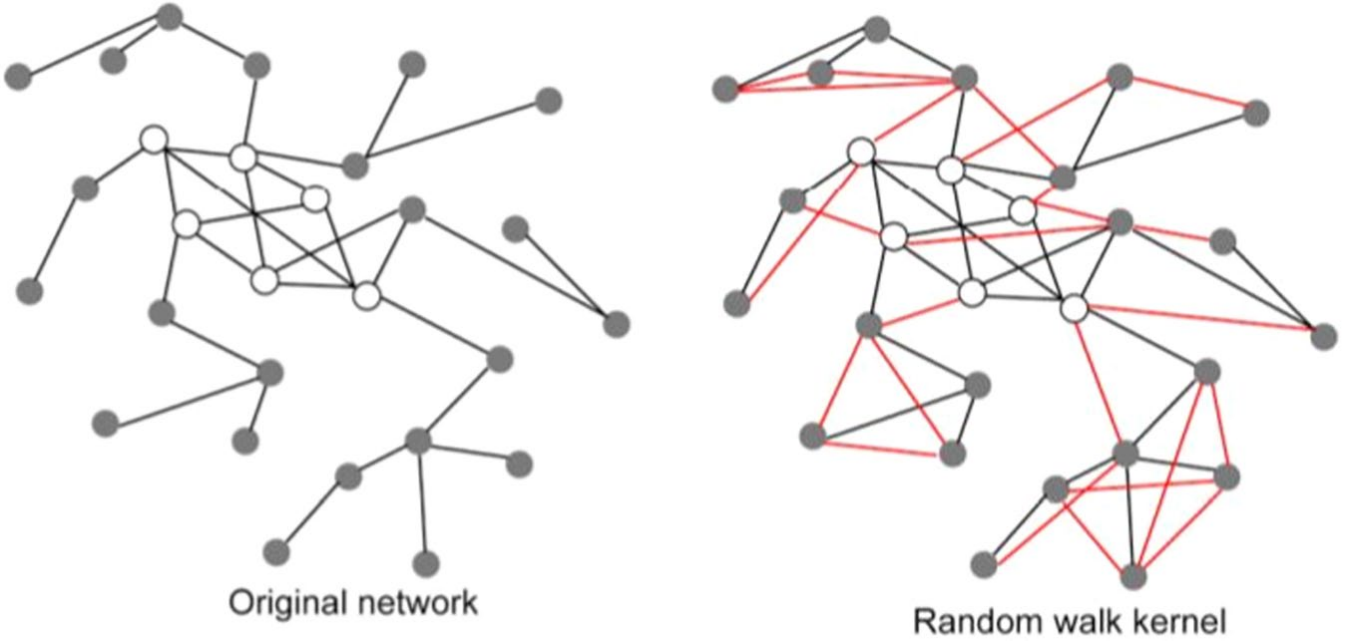
The graph kernel induces a novel similarity measure between patients. The original network (left side of the figure) is constructed using a suitable similarity measure between patients (e.g. the Pearson correlation). The *p* − *step* random walk kernel “enriches” the original graph by modifying the weights of the original network and by possibly adding new edges (coloured in red, graph in the right side): if two nodes are indirectly connected through a path of length equal at most to *p*, a new edge is added between them, and its strength depends on the (possible) multiple paths and the weights of the edges along the paths. In other words the graph kernel implicitly induces a new non linear similarity measure between patients that takes into account the topology of the network and the initial similarity between patients of the original network.
4. **Filtering of the kernel matrix** The resulting kernel matrix ***K*** is usually very dense, and its sparsification is a crucial step that allows to retain only relevant edges, thus reducing the noise introduced by very poor similarities between patients. Unfortunately, simply setting an “a priori” threshold, as usually done in other experimental contexts^33,38,39^, leads to poor results. To overcome this problem, we select a threshold *τ* to filter the edges using a very efficient leave-one-out technique that maximizes a pre-selected performance measure (e.g. the Area Under the Receiving Operating Characteristic - AUC) on the training set (see Section “Implementation of *P-Net*” for more details).
5. **Ranking of patients with score functions** The *score functions* associate a score to each node of the network on the basis of the labeling of its neighborhood and the weights of its incoming edges (Table 1). *S*_*AV*_, *S*_*NN*_ and *S*_*kNN*_ (Eqs. 1, 2 and 3), derived as described in^33,34^, compute their score respectively considering the average, the nearest and the *k* nearest labeled neighbours. While these scores exploit only weights coming from the labeled neighbours, the remaining score functions *S*_*TOT*_, *S*_*Diff*_ and *S*_*Dnorm*_ (Eqs. 4, 5, and 6) allow us to exploit the information that comes from the whole neighborhood of the investigated node *i*, including both nodes *i* ∈ *V*_*C*_ and *i* ∉ *V*_*C*_. In this way the labeling of each node depends on both “positive” and “negative” annotations of the neighborhood nodes.

**Table 1:** Score functions. Elements of ***K*** are represented by *k*_*ij*_, and its *i*^*th*^ row by ***K***_*i*_, while positive integers *i*, *j* represent nodes (patients); *V*_*C*_ ⊂ *V* represents the set of “positive” patients, i.e. patients associated with the phenotype or clinical outcome *C* of interest.

Score Name	Formula
*Average*	${S}_{AV}(i,{{\boldsymbol{K}}}_{i},{V}_{C})=\frac{1}{| {V}_{C}| }{\sum }_{j\in {V}_{C}}{k}_{ij}\quad \quad (1)$
*Nearest Neighbour*	${S}_{NN}(i,{{\boldsymbol{K}}}_{i},{V}_{C})=\mathop{max}\limits_{j\in {V}_{C}}{k}_{ij}\quad \quad (2)$
*k-Nearest Neighbour*	$$\begin{array}{ccc}{S}_{kNN}(i,{{\boldsymbol{K}}}_{i},{V}_{C}) & = & \frac{1}{|{I}_{k}(i)|}{\sum }_{j\in {I}_{k}}{k}_{ij}\\ {\rm{w}}{\rm{h}}{\rm{e}}{\rm{r}}{\rm{e}}\,{I}_{k}(i) & = & \{j|j\in {V}_{C}\wedge {\rm{r}}{\rm{a}}{\rm{n}}{\rm{k}}({k}_{ij})\le k\}\end{array}\,\,(3)$$
*Total*	${S}_{TOT}(i,{{\bf{K}}}_{i},{V}_{C})=\frac{{\sum }_{j\in {V}_{C}}{k}_{ij}}{{\sum }_{j\in {V}_{C}}{k}_{ij}+{\sum }_{j\in V\backslash {V}_{C}}{k}_{ij}}\quad \quad (4)$
*Differential*	${S}_{Diff}(i,{{\bf{K}}}_{i},{V}_{C})={\sum }_{j\in {V}_{C}}{k}_{ij}-{\sum }_{j\in V\backslash {V}_{C}}{k}_{ij}\quad \quad (5)$
*Differential normalized*	${S}_{Dnorm}(i,{{\bf{K}}}_{i},{V}_{C})=\frac{{\sum }_{j\in {V}_{C}}{k}_{ij}-{\sum }_{j\in V\backslash {V}_{C}}{k}_{ij}}{{\sum }_{j\in {V}_{C}}{k}_{ij}+{\sum }_{j\in V\backslash {V}_{C}}{k}_{ij}}\quad \quad (6)$

*P-Net* provides a score to rank patients, but by setting a threshold *τ* we can obtain a classifier: patients having a score larger than *τ* are classified as positive and the others as negative. In our experiments, we set the threshold according to the predicted best accuracy by a fast internal leave-one-out procedure on the training set (see Section “Implementation of *P-Net*”), and then we applied the selected optimal threshold on the test set. Additional information about *P-Net* and its implementation are available in the next Section and in the Section “Efficient implementation of P-Net” in the Supplementary Information.

### Implementation of *P-Net*

The analysis of the generalization performances of *P-Net* and the selection of the network threshold *τ* (see “Filtering of the kernel matrix” in the “Methods” Section), can be performed through cross-validation or leave-one-out (LOO) procedures. In particular, to obtain an unbiased evaluation of the generalization performance and an unbiased network filtering, we performed a double leave-one-out procedure: (a) an internal LOO to select the network threshold *τ*; (b) an external LOO to estimate the generalization capabilities of the algorithm.

Unfortunately, the classical implementation of the double LOO requires to run the algorithm *n*^2^ times, where *n* is the number of patients. To avoid this computational burden, we propose an efficient implementation of the *P-Net* LOO, that requires only a unique run across patients.

**Theorem 1**: *Having a kernel matrix* ***K*** *obtained from the weighted adjacency matrix* ***W*** *of a graph* *G* = <*V*, *E* >, *with vertices* *v* ∈ *V* *denoted with* *i* ∈ { 1, …, |*V*|}, *and* *N*(*i*) = {*j* | *j* ∈ *V*, *k*_*ij*_ > 0} *the neighbourhood of node* *i* *in the graph* *G* *transformed according to the kernel* ***K***, *when a leave-one-out procedure is applied with the* *P-Net* *algorithm*, *the following fact holds*:

$${k}_{ii}=0\;\iff \;i\ is\ left\ out.$$

*Proof:*

I. *k*_*ii*_ = 0 ⇒ node *i* is left out.

If ∀ *i,* *k*_*ii*_ = 0, one of two possible conditions holds:

1. if *i* ∉ *N*(*i*), *k*_*ii*_ is not included in the computation of the score function *S*(*i*), where *S* is one of the scores functions described in Section “Methods”.
2. if *i* ∈ *N*(*i*), *k*_*ii*_ is considered in the score function, but by hypothesis *k*_*ii*_ = 0

In both cases the score of the node *i* is computed independently of the labeling of the node *i*, that is, in other words, the node *i* is left out.

II. *k*_*ii*_ = 0 ⇐ node *i* is left out.

If *i* is left out, even if *i* ∈ *N*(*i*), *k*_*ii*_ is not used in the computation of the score *S*. Equivalently we can set *k*_*ii*_ = 0 and then we can use *k*_*ii*_ in the computation of *S*(*i*), since it has no effect on the computation of the score.□

**Figure 3:**
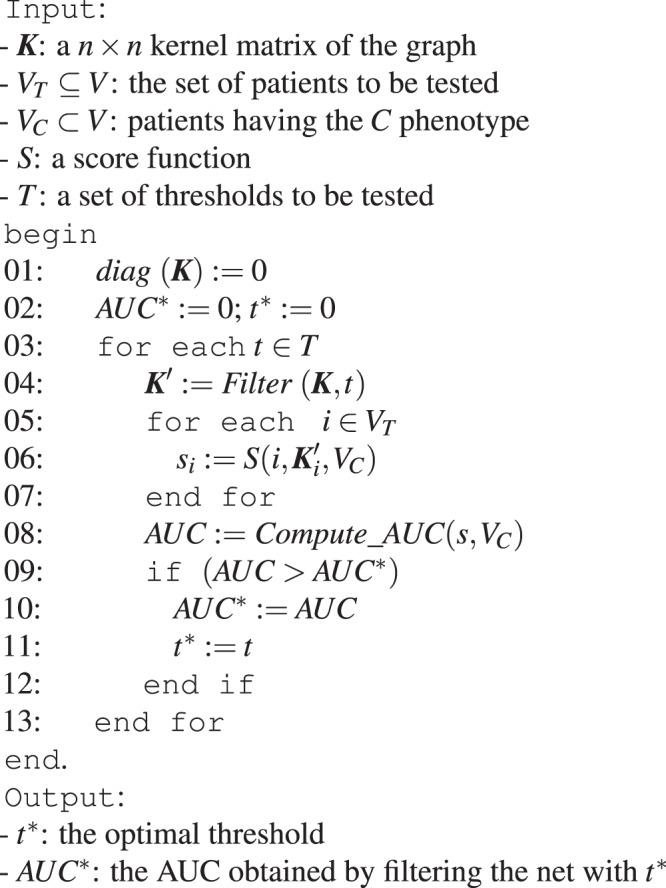
A *P-Net* procedure to find the “optimal” filtering threshold *τ*.

The intuition behind the efficient LOO implementation is that instead of moving away an example/node at each round of the LOO procedure and recomputing the score for all the nodes of the network, we can equivalently set to zero the self-loop edge of that node. Indeed the information about the labeling of the held-out node *i* is used only in step 5 of the algorithm (see “Ranking of patients with score functions” in the “*P-Net* algorithm” section), and by setting *k*_*ii*_ = 0 we automatically exclude that node by the computation of its score, i.e. we hold-out that node from the training set. As a consequence of the above theorem, to perform the LOO it is sufficient to set to 0 the diagonal of ***K*** and then run one time *P-Net* across all the nodes of the graph *G*. For instance, to select the optimal threshold *τ*, we can simply set to zero the diagonal of the kernel matrix ***K*** and then apply a unique run of P-Net (Fig. 3).

The use of the efficient LOO *P-Net* procedure for evaluating the generalization performance of the algorithm and to select the optimal threshold of the network are described in detail in the Section “Efficient implementation of *P-Net*” in the Supplementary Information.

### Accession codes

The datasets from Melanoma, Ovarian, Breast, Colorectal and Colon cancer are all available from the Gene Expression Omnibus database (accession numbers GSE53118, GSE26712, GSE2990, GSE17536, GSE17538). The Pancreatic cancer dataset is available from ArrayExpress Archive (accession number E-MEXP-2780).

## Results

We compared *P-Net* with both supervised inductive methods and semi-supervised network-based algorithms for outcome/phenotype prediction. Among supervised inductive methods we selected Support Vector Machines (SVMs) and Random Forests since they showed superior performances for these tasks^40–42^.

We also considered an approach that applies as a first step a network-based method (Net-Rank^43^), as well as other univariate feature selection methods, to select the most relevant features, and as a second step a supervised algorithm to predict the outcome using the previously selected features^26^. Net-Rank enhances the Google Pagerank-learning approach^44^ by using Laplacian regularization and an additive margin: for PageRank the nodes are web pages and the edges are hyperlinks between them; for NetRank the vertices of the network are genes and the edges are relationships between them (e.g. protein-protein or transcription factor-target interactions). Then a Support Vector Machine is trained with the selected features/biomarkers to predict the outcome of patients^26^.

Finally, we compared our method with an approach developed by Park *et al*.^19^. Similarly to *P-Net*, this method applies a semi-supervised learning algorithm on the similarity graph of patients to rank and classify them according to a specific phenotype/outcome. However this method differs from *P-Net* since it adopts a solution in closed form to minimize the cost function where both the mislabeling of the patients (the nodes of the graph) and the discrepancy between predicted labels of connected nodes in the graph are jointly minimized. Instead, in our semi-supervised approach, a graph kernel is firstly applied to capture the overall topological characteristics of the graph, and then a score function is applied over the transformed graph to rank patients.

We compared P-Net with each of the above cited methods^19,26^ by using the same experimental set-up, metrics and datasets used by each of them. We also applied the same filtering approach adopted in the first step of the *P-Net* algorithm (i.e. a classical t-test) to select the genes significantly associated with the patients’ phenotype/outcome, and we performed gene set enrichment analysis^45^ to uncover pathways and Gene Ontology terms overrepresented in the set of selected genes, to characterize the underlying disease mechanisms.

The data sets used in the experiments are publicly available and are schematically described in the next section.

### Datasets description

We applied our novel *P-Net* method on several microarray gene expression cancer data sets.

The Pancreatic ductal adenocarcinoma data set from Winter *et al*.^26^ includes 30 patients, split into two groups (*good prognosis* and *poor prognosis*) according to their survival time (threshold set at 17.5 months, as in the original paper^26^). The Breast, Colorectal and Colon cancer patients were originally analyzed by Park *et al*.^19^. Breast cancer data are composed of 189 invasive breast cancer patients, divided into the classes “high” and “low” risk of recurrence; Colorectal cancer data includes 177 patients and Colon cancer 213, both divided into the groups “recurrence”, “no recurrence”. All these data sets include also “unlabeled” patients, i.e. patients having no prognostic or phenotype associated information. Table 2 shows a high-level summary of the data, and further details are provided in “Datasets” Section of the Supplementary Information.

**Table 2:** Summary of all the datasets employed in this work. “Positive” patients are those with *poor prognosis* or *high risk of recurrence*.

Dataset	Accession number	Database	Labeled patients	Unlabeled patients
Pancreatic	E-MEXP-2780	ArrayExpress	30 (15 positives)	0
Breast	GSE2990	GEO	125 (49 positives)	64
Colorectal	GSE17536	GEO	145 (36 positives)	32
Colon	GSE17538	GEO	181 (49 positives)	32

### Analysis of Breast, Colorectal and Colon cancer patients

#### Experimental set-up

We compared *P-Net*, using Breast, Colorectal and Colon cancer data, with Park *et al*. semi-supervised graph-based method that minimizes both the classification error and the “internal consistency” of the predicted labeling^19^. Moreover we compared *P-Net* on the same data sets with three other supervised inductive methods, i.e. SVM, Random Forest and Naive Bayes. In the above mentioned experiments we employed the same experimental set-up, based on 10-fold cross-validation, as proposed in^19^. With these data sets *P-Net* achieved the best results with a Random Walk Kernel 1-step and the differential or the differential normalized score function (Table 1). More details about the experimental set-up are available in the *Section “Experimental set-up for the analysis of Breast, Colorectal and Colon cancer patients”* in Supplementary Information.

#### Results

**Figure 4:**
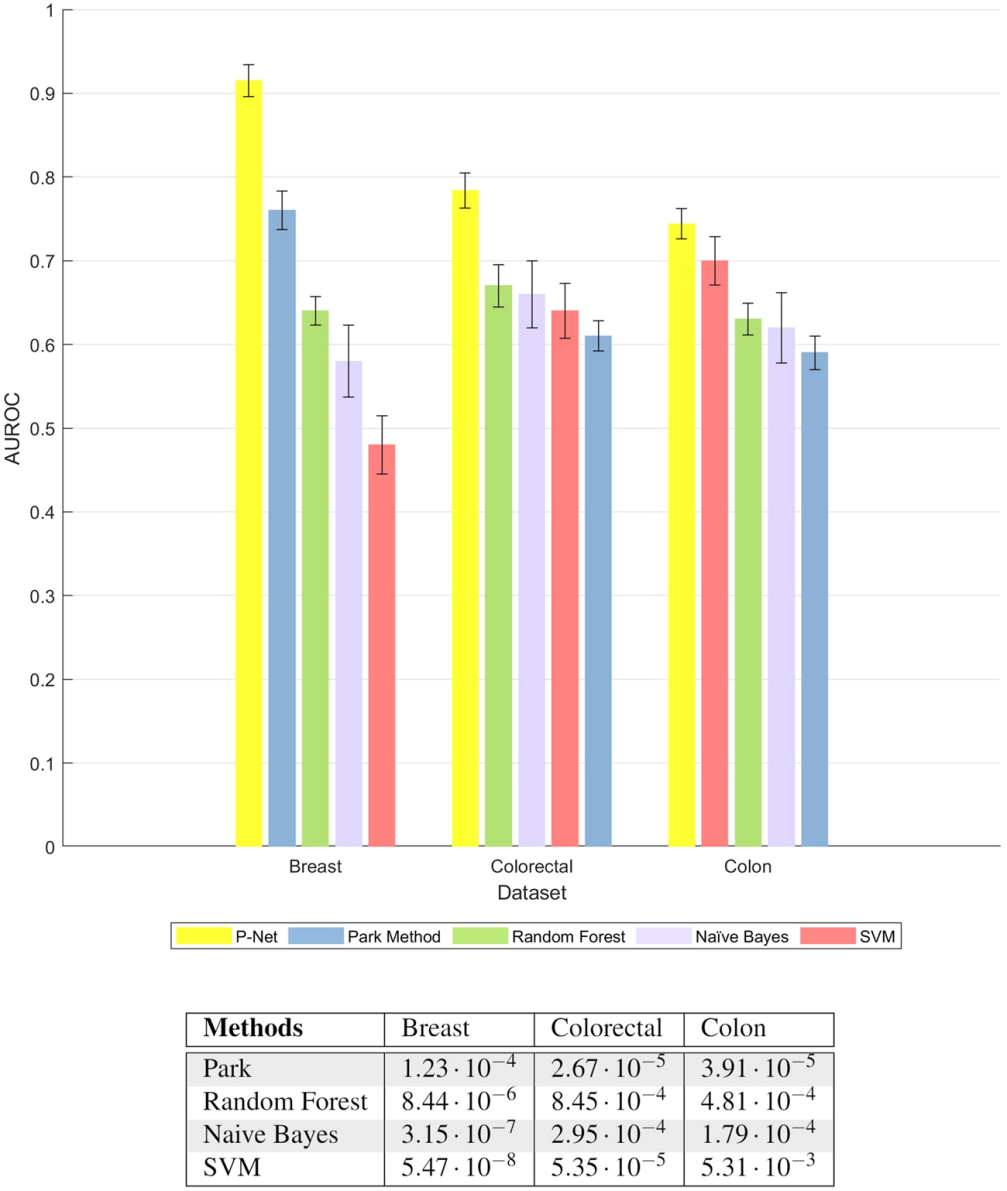
Comparison of the average AUROC values obtained by *P-Net* with the Park’s, Random Forest, Naive Bayes, and SVM methods over three datasets. AUROC results are averaged across 15 rounds of the 10-fold cross-validation. Top: average AUROC with error bars indicating standard deviation (for each dataset, bars are sorted according to decreasing performance). Bottom: statistical comparison of *P-Net* with the other methods. P-values are computed by one tail Wilcoxon rank sum test across 15 repetitions of 10-fold cross-validation. Rows refer to the methods compared with *P-Net*; columns to Breast, Colorectal and Colon cancer data.

*P-Net* outperforms the other methods in terms of the Area Under the Receiving Operating Characteristic (AUROC) (Fig. 4). Moreover the results are statistically significant, according to the one-tail Wilcoxon rank sum test with Bonferroni correction: indeed by applying the statistical test to the repeated 15 cross-validation we always obtain a p-value lower than 0.01 (Table at the bottom of Fig. 4).

Also, the accuracy and the sensitivity of *P-Net* are competitive with the other compared algorithms (see Table S2 Supplementary Information for more details).

### Analysis of Pancreatic ductal adenocarcinoma patients

We compared *P-Net* with a method that applies linear Support Vector Machines trained on biomarkers selected through both a network-based method (NetRank) and other simpler univariate feature selection methods^26^. We did not use non-linear SVMs since in^26^ the authors reported that more complex kernels did not lead to better results.

#### Experimental set-up

Winter *et al*.^26^ used NetRank and a series of univariate feature selection methods: fold change, t-statistic, Pearson and Spearman rank correlation coefficients, SAM (Significance Analysis of Microarray) method^46^, and random selection of genes as control, to select the genes most correlated with the survival time of a patient. Then the selected top ranked genes were used to train a Support Vector Machine (SVM) in order to predict patients with poor (PP) or good (GP) prognosis.

The generalization performances were evaluated through a Monte Carlo cross-validation technique (MCCV). More precisely we randomly split the data in training and test set 1000 times. At each round of the splitting procedure, we performed feature selection through the t-test using only the data of the training set. Then we constructed a network of patients using only the selected genes and we filtered the edges according to the P-Net accuracy on the training set by the efficient leave-one-procedure described in the Section “Implementation of P-Net”. The score threshold to optimally separate poor and good prognosis patients is computed on the training set and the generalization performances are finally evaluated on the separated test. A detailed step by step description of the experimental set-up is available in the Section “Experimental set-up with Pancreatic cancer data” in Supplementary Information.

#### Results

**Figure 5:**
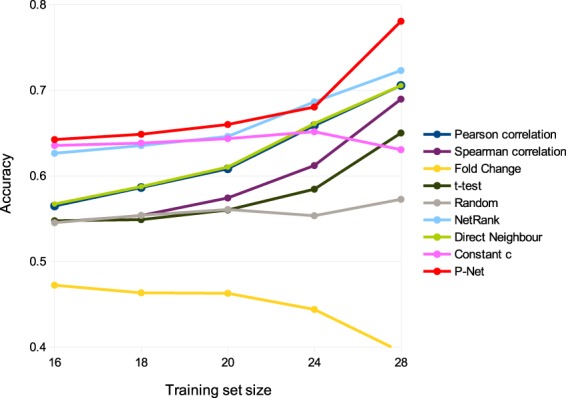
Comparison of the accuracy of *P-Net* and a SVM trained with NetRank and other feature selection methods on the Pancreatic cancer dataset. *P-Net* results are shown in red, SVM with NetRank in cyan, the other colors represent the results of the SVM with other feature selection methods (Pearson and Spearman correlation, Fold change, t-test), with random selection and with two other variants of the NetRank algorithm (Direct Neighbour and Constant c). The x axis reports the number of patients in the training set; y axis reports the accuracy.

*P-Net* used the Random Walk Kernel, and the model with *p* = 8 steps, the Nearest Neighbour score function and a set of genes selected according to the t-test achieved the best results. Fig. 5 shows the results (average accuracy) for a different number of patients in the training set (from 16 to 28). *P-Net* achieves better results than the other methods for each training set size, except with 24 patients, where it obtains the second best result. In all cases the difference is statistically significant (t-test at 0.05 significance level). Detailed results showing average accuracy and standard error of the mean for all assessed methods are available in Table 3.

**Table 3:** Comparison of the methods applied to the Pancreatic cancer dataset. The best accuracy value achieved for each training set is highlighted in bold.

Training set size	16	18	20	24	28
Method	Accuracy (Standard Error of the Mean)				
Pearson correlation	56.47%	58.63%	60.82%	65.87%	70.55%
	(0.39%)	(0.41%)	(0.45%)	(0.60%)	(0.97%)
Spearman correlation	54.57%	55.35%	57.41%	61.20%	68.95%
	(0.38%)	(0.41%)	(0.47%)	(0.58%)	(1.02%)
Fold Change	47.21%	46.33%	46.27%	44.38%	39.45%
	(0.34%)	(0.37%)	(0.42%)	(0.53%)	(1.04%)
t-test	54.74%	54.88%	56.00%	58.43%	65.00%
	(0.39%)	(0.42%)	(0.47%)	(0.61%)	(1.10%)
Random	54.51%	55.38%	56.06%	55.33%	57.25%
	(0.40%)	(0.40%)	(0.46%)	(0.58%)	(1.08%)
NetRank	62.63%	63.53%	64.60%	**68.63%**	72.30%
	(0.34%)	(0.38%)	(0.43%)	(0.56%)	(0.99%)
Direct Neighbour	56.70%	58.73%	61.02%	66.10%	70.55%
	(0.39%)	(0.41%)	(0.46%)	(0.60%)	(0.97%)
Constant *c*	63.54%	63.82%	64.34%	65.15%	63.05%
	(0.32%)	(0.33%)	(0.38%)	(0.56%)	(1.04%)
P-Net	**64.23%**	**64.85%**	**65.99%**	68.03%	**78.05%**
	(2.02%)	(2.23%)	(2.38%)	(3.38%)	(7.38%)

**Figure 6:**
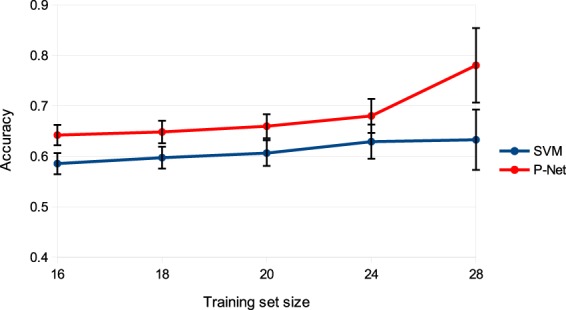
Comparison of SVM and *P-Net* after the selection of the top ranked 1000 genes through t-test. The vertical bars represent the Standard Error of the Mean (SEM) of the accuracy across 1000 repetitions of the hold-out procedure.

We performed also an additional experiment to understand whether a larger set of biomarkers selected using a t-test can enhance the performance of the SVM with respect to *P-Net*. Results show that, even when selecting a relatively large number of biomarkers associated with the poor prognosis (1000 genes), *P-Net* significantly outperforms the SVM for any size of the training set (Fig. 6).

In the Section “Summary of P-Net results” in the Supplementary Information we report the detailed results obtained by *P-Net*, including the average AUROC, AUPRC, F1-score and accuracy for each data set. Results show that, as expected, *P-Net* is more stable when relatively large data sets are used.

### Assessment of the statistical significance of the patients ranking

For all the considered data sets, we applied a non parametric test based on random shuffling of the labels to assess the statistical significance of the prediction results^47^. More precisely we repeat 10000 times a random permutation of the labels, and at each iteration of the shuffling we compare the AUC obtained by *P-Net* with the “true labels” with that obtained with the randomly permutated labels. The estimated p-value is the frequency by which the AUC computed with the shuffled labels is larger than that computed with the true labels. More details are available in the Section “A non parametric test to validate patient ranking” in the Supplementary Information. Table 4 shows that the computed p-values are always less than 0.05, confirming that the ranking is not due by chance.

**Table 4:** Validation of *P-Net* patients’ ranking using a non parametric test on the different datasets. Results of the random label permutation test to assess the significance of patients ranking. RWK stands for Random Walk Kernel, Diff. score for Differential score, Dnorm score for Differential normalized score. For the Pancreatic cancer dataset, results with different training set sizes are shown.

Dataset	*P-Net* methods	p-value (ranking)
Pancreatic - 16 patients	RWK8, NN score	0.0010
Pancreatic - 18 patients	RWK8, NN score	0.0060
Pancreatic - 20 patients	RWK8, NN score	0.0120
Pancreatic - 24 patients	RWK8, NN score	0.0060
Pancreatic - 28 patients	RWK8, NN score	0.0170
Breast	RWK1, Dnorm score	0.0000
Colorectal	RWK1, Diff. score	0.0490
Colon	RWK1, Diff. score	0.0440

### Characterization of genes and pathways involved in patients’ outcome

Linking predictions to the underlying disease mechanisms is of paramount importance, and to this end we applied classical gene set enrichment analysis to uncover pathways associated with patients’ phenotype and outcome, starting from the set of genes selected as differentially expressed at the first step of the *P-Net* algorithm. We excluded from the analysis the pancreatic cancer patients, due to the relatively small number of available examples. More precisely, we selected the genes significantly associated with phenotype/outcome using the classical t-test with Benjamini-Hochberg correction^48^ at *p*-*value* < 0.05 for colorectal and colon cancer and with Bonferroni correction with *p*-*value* < 0.001 for breast cancer. The more restrictive Bonferroni correction is needed to obtain a relatively reduced set of differentially expressed genes in breast cancer. We repeated the selection using only genes of the training sets across multiple iterations of the 10-fold cross-validation procedure, and we finally selected only those genes robustly selected at least 70% of times. Each set of robustly selected genes was further analysed using the conditional hypergeometric test to find overrepresented GO:BP terms (*p*-*value* < 0.05) and the standard hypergeometric test to discover overrepresented KEGG pathways (*p*-*value* < 0.05), as implemented in the R package GOstats^45^. Since the lists of overrepresented GO terms for breast and colorectal cancer were very large, we used REVIGO^49^, a clustering algorithm based on semantic similarity measures, to obtain a representative subset of more interpretable terms.

Additionally, the obtained gene lists were analyzed by Ingenuity Pathway Analysis (IPA, QIAGEN Inc., https://www.qiagenbioinformatics.com/products/ingenuity-pathway-analysis) software^50^ (*p*-*value* < 0.05) in order to further investigate enriched signaling and metabolic pathways related to our selected genes.

Results show that we found genes and pathways known to be associated with breast, colon and colorectal cancer, as summarized below. Full lists of the associations, supported by results documented by literature are available in Supplementary Information (Tables S3–S8, Fig. S6).

#### Breast cancer

364 genes were selected by t-test and used for further functional analysis. Among overrepresented KEGG pathways we found “Proteasome” which is a complex dedicated to protein catabolism involved in several cellular functions (e.g. regulation of cell cycle, signaling pathways, stress signaling, apoptosis). Recent studies suggested that deregulation of the ubiquitin-proteasome pathway may have a permissive role in breast cancer development, and drugs which target this pathway are currently in clinical trial^51^. From our analysis the pathways “Protein processing in endoplasmic reticulum” and “Oxidative phosphorylation” resulted overrepresented and associated with breast cancer in literature. Endoplasmic reticulum guarantees that only correctly folded proteins reach their final destination in the cell while unfolded/misfolded ones are degraded by the proteasome. However, endoplasmic reticulum stress (ERS), i.e. the accumulation of unfolded/misfolded proteins in the reticulum, may arise due to glucose deficiency, hypoxia, calcium imbalance and oxidative stress. ERS is associated with tumor development and metastasis in breast cancer since the high cell proliferation rate in tumors causes hypoxia, nutrients starvation and higher ROS production^52,53^. In particular, increased ROS production in cancer cells arises from oxidative phosphorylation, oxygen metabolism and NADPH oxidase functions leading to oxidative stress, described also in breast cancer^54^.

Considering GO:BP terms, we found some common biological processes known to be involved in tumor development, such as “cell cycle phase transition”, “apoptotic signaling pathway” and “signal transduction by p53 class mediator”. Defects in the cell cycle checkpoints are associated with breast cancer molecular subtypes^55^ while apoptosis escape is one of the hallmarks of cancer^56^. p53 is a tumor suppressor able to avoid cancer development through block of the cell cycle, programmed cell death, repair of damaged DNA and senescence. p53 is mutated in 20-30% of breast cancers and it is generally silenced by loss of upstream/downstream mechanisms^57^. Another interesting term is “cellular oxidant detoxification” which is consistent with the presence of oxidative stress in breast cancer cells^58^.

Finally, from IPA analysis mTOR-p70S6K signaling resulted enriched in the Top Canonical Pathways, in agreement with its frequent deregulation found in breast cancer, often associated to drug resistance^59^. The analysis also revealed that “Cell Death and Survival” and “Cellular Growth and Proliferation” are the molecular and cellular functions most significantly overrepresented in the recurrence-associated genes (207 and 139 out of 364 molecules, respectively). These findings are consistent with GO:BP hypergeometric test and the selected genes encoding for cyclins (CCNA2, CCND2), enzymes involved in ubiquitination (UBE2D2, UBE2L3, UBE2N) and the transcription factor STAT1. These results are in line with those obtained by other groups which analyzed GSE2990 dataset^60–62^. In particular, Sotiriou and coworkers reported that genes predicting high risk of recurrence are mainly involved in cell cycle regulation and proliferation^60^. Of note, STAT1 was identified as a breast cancer recurrence gene also by Park and colleagues^62^ and it was associated to resistance to endocrine therapy and to distant metastasis-free survival by Huang and others^61^.

#### Colon and colorectal cancer

A total of 55 genes were found associated with recurrence in colon and colorectal cancer. In colon cancer, KEGG pathway analysis highlighted dysregulations in few metabolic pathways, such as “Fatty acid biosynthesis”, “Propanoate metabolism” and “Pyruvate metabolism”. Lipid accumulation was observed in many cancer types (e.g. brain, breast, ovarian and colorectal cancers), caused by an imbalance between fatty acid biosynthesis and *β*-oxidation, where lipids are used by fast growing cells to support various activities like membrane formation and signaling. Abnormal expression of genes involved in fatty acid metabolism was found by different studies in correlation with metastasis, drug resistance and relapse^63^. Notably, many GO:BP terms found in our analysis are consistent with aberrant lipid metabolism (“malonyl-CoA biosynthetic process”, “negative regulation of fatty acid beta-oxidation”, “carnitine shuttle”, “positive regulation of lipid storage”, “negative regulation of fatty acid metabolic process”, “regulation of fatty acid oxidation”, “acetyl-CoA metabolic process”, “regulation of lipid catabolic process”, “sterol biosynthetic process”, “fatty acid transport”). Considering “Propanoate metabolism” and “Pyruvate metabolism”, most tumor cells highly depend on aerobic glycolysis rather than mitochondrial oxidative phosphorylation to obtain energy, known as “Warburg effect”, and this disregulates the above mentioned metabolic paths^64^. Moreover, GO:BP analysis revealed multiple terms related to “interleukin mediated signaling pathway” (i.e. IL-2, IL-7, IL-9, IL-12 and IL-15). For instance, Kuniyasu *et al*.^65^ associated the production of IL-15 in colon cancer cells with proliferation, resistance to apoptosis, metastasis and angiogenesis.

In colorectal cancer, pathway analysis showed that “Pathogenic Escherichia coli infection” is associated with the disease. Indeed, in literature some strains of E. coli are known to produce a genotoxic metabolite (colibactin) that alkylates DNA *in vivo* and contributes to the development and progression of this tumor^66^. Among biological processes consistently overrepresented in our analysis, we found GO:BPs related to the *β*-catenin destruction complex (i.e. “beta-catenin destruction complex disassembly”, “beta-catenin destruction complex assembly”, “catenin import to nucleus”), nitric-oxide biosynthesis (“positive regulation of nitric oxide biosynthetic process”, “positive regulation of nitric oxide biosynthetic process”, “nitric oxide production involved in inflammatory response”), inflammasome (“positive regulation of NLRP3 inflammasome complex assembly”, “pyroptosis”). The *β*-catenin destruction complex was recently found disrupted in colorectal cancer^67^, possibly enabling the migration of *β*-catenin to the nucleus and the subsequent transcription of target genes. Aberrant stabilization of the *β*-catenin due to mutations in the destruction complex was associated with various cancers^68^. Regarding the biosynthesis of nitric-oxide, colorectal cancer is strongly associated with chronic inflammation and nitric-oxide produced by NOS2 is associated with the initiation and progression of the disease. Moreover, over-expression of NOS2 is correlated with poor outcome^69^. Finally, the activation of NLRP3 inflammasome, with consequent production of IL-1*β*, IL-18 and procaspase-1 activation which induces pyroptosis (i.e. a form of programmed cell death), showed an anti-tumoral effect preventing/inhibiting colorectal cancer development^70^.

Functional annotation of selected genes by IPA revealed that 13 out of 54 genes are closely associated to “Cell Growth and Proliferation” and “Cell Death and Survival” (AMER1, ATOH7, CSPP1, CTNNB1, DCD, FGF4, JAK1, NLRC4, PRF1, SLC29A2, SLC4A1, SP7, TRL4). The top canonical pathways enriched for these genes were “Epithelial-Mesenchymal Transition (EMT)”, “Inflammasome” and “iNOS signaling” (see Supplementary Fig. S6). Some pathways were detected by both GOstats and IPA. In particular, EMT pathway has been strongly associated with the invasive and metastatic phenotype in colon cancer^71^. Among the deregulated genes involved in the EMT signaling, CTNNB1, as well as JAK1 play key roles in colon cancer progression: CTNNB1 through the activation of Wnt/*β*-catenin signaling, and JAK/STAT pathway by regulating cell survival and proliferation, differentiation and migration^72^. Our findings are in line with previous studies showing that CTNNB1 and JAK family members are colon cancer recurrence-specific genes and prognostic biomarkers^62,72–74^. Notably, we also identified NLRC4 gene, a member of NOD-like receptors in the “Inflammasome Pathway”, as a novel gene related to colon cancer recurrence. Previous studies have shown that NLRC4 takes part in inflammation-induced colon cancer tumorigenesis and participates in anti-apoptotic pathways downstream of p53^75^. In addition, a recent study showed that NLRC4 mediates M2 TAM infiltration and angiogenesis through VEGF production in metastatic colon cancer^76^.

Summarizing, the genes and pathways selected in the first step of *P-Net*, are not only fundamental to achieve good phenotype/outcome predictions, but are also related to the biological mechanisms underlying the disease.

### Visualization of the P-Net graph

Supervised inductive models in most cases are “black boxes” not suitable for interpreting and explaining the obtained results. On the contrary *P-Net* constructs a graph of patients, explicitly showing their biomolecular relationships and embedding at the same time the predicted scores in the graph. The graph can be easily shown to the user to provide a visual clue of the relationships between patients by means of a graph visualization tool; moreover, the predicted associated phenotype can be properly visualized by exploiting a range of colors and shapes. The user is thus enabled to conduct a direct visual inspection of the network, which might allow him/her to either uncover hidden relationships/insights about the phenotypic and/or biomolecular characteristics of the samples (e.g. disclosing interesting mismatches among similar patients, close in the graph, but with different predicted phenotype), or might suggest stratifications of patients on the basis of their similarity or predicted phenotype.

**Figure 7:**
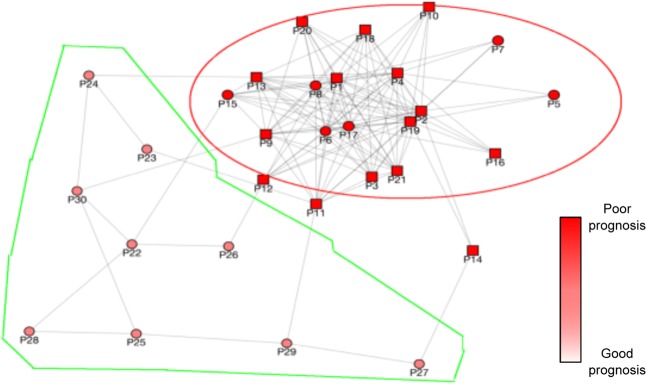
Pancreatic cancer graph constructed by *P*-Net. Square nodes represent “poor prognosis”, while circles “good prognosis” patients. The colouring of the nodes is related to the predicted prognosis.

As an example of this opportunity, Fig. 7 shows the visual representation of the graph constructed by *P-Net* with the Pancreatic ductal carcinoma data set obtained by adapting to our context a graph visualization tool developed in our laboratory for visualizing biomolecular interactions (the tool is detailed in Perlasca *et al*.^77^). In the figure the patient ground truth is shown by the node shapes (circles for good prognosis patients, and rectangles for poor prognosis patients) while the node color shows the prognosis predicted by *P-Net*. The graphical representation shows that the group of good prognosis patients (highlighted in green) are all correctly predicted by *P-Net*. A second group (highlighted by a red ellipse) includes poor prognosis patients predicted by our method, some of which are wrongly classified. However, though being labeled as “good prognosis”, the “misclassified patients” are really borderline patients: their survival time is very close to the cut-off of 17.5 months selected in^26^ to separate good from poor prognosis patients. As another example, Fig. 5 in Supplementary Information visualizes the network of Breast cancer patients.

Thus, the graph visualization, eventually coupled with graph clustering algorithms^78^ and different visualization perspectives and interactions, can allow domain experts to identify interesting visual explanations of the achieved classification, which would not be so evident in a textual representation of the graph. Different approaches have been proposed along this direction, such as^77^ in the context of biomolecular interactions and^79^ for the visualization of phenotype similarities. These tools offer different functionalities for clustering graph nodes according to different similarity measures, for changing the color and the shape of the nodes according to different parameters, for changing the layout perspectives and pointing out different graph properties. All these functionalities aid experts to uncover hidden information represented in the graph and to obtain further clues about different patients.

## Discussion and Conclusion

*P-Net* is a semi-supervised transductive approach where the genetic similarities between patients guide the process of outcome prediction and provide a graph representation of the biomolecular similarity between patients. *P-Net* analyses the biomolecular profiles of patients by firstly constructing a similarity graph between them and then applying a graph kernel, which extends the notion of similarity between patients and exploits the global topology of the graph to discover novel relationships between them. Finally, *P-Net* ranks patients with respect to the phenotype/outcome under study.

Our experimental results with different cohorts of cancer patients show that this approach is competitive with both network-based and inductive models for outcome prediction. The fast implementation of the *P-Net* leave-one-out procedure, together with the low spatial and time computational complexity of the method (see Section “Implementation of P-Net” and Supplementary Information), allow an easy application to actual clinical and biomolecular data using off-the-shelf desktop or laptop computers. It is worth noting that by combining different kernels and score functions we can obtain different variants of *P-Net*. From this standpoint *P-Net* can be considered as an algorithmic scheme from which specific learning algorithms can be derived by choosing a specific graph kernel and score function.

Several works showed that the outcome results are often not statistically significant and basically due by chance^80,81^. To address this problem, by using a non parametric test based on random shuffling of the labels, we showed that *P-Net* ranking of patients is not due by chance, but on the contrary is significantly related to the outcome.

Importantly, *P-Net* is not only able to predict the phenotype/outcome, but can also construct a graph of patients that can be visualized to point out the similarities between their biomolecular profiles and, more in general, the relationships between them. In this way we can detect, by visual inspection, closely related patients as possible subtypes of a given pathology, or we can identify an incorrect prognosis for a specific patient or for possible “outlier patients”. Finally the filtering step of *P-Net* can be used not only to improve its prediction performance, but also to link predictions to the underlying disease mechanisms, by uncovering genes and pathways associated with patients’ outcome/phenotype.

It has been shown that the integration of different kinds of data produces synergies between data that can improve the prediction performances^82–84^. To this end we need at first to construct patient similarity networks using similarity measures appropriate for the data at hand. For instance, Pai and Bader for low dimensional data (e.g. clinical data) proposed simple measures such as the normalized and average normalized similarity^20^. For patient profiles characterized by a medium size, such as mRNA data, protein expression, miRNA, the same authors proposed correlation-based measures (e.g. Spearman, Pearson with and without exponential scaling, euclidean or more in general p-norm distances) with or without an initial pre-filtering (using e.g. univariate statistics or machine learning based feature selection methods). For very high dimensional data (such as genotypic or epigenomic data), a pre-filtering step is mandatory to reduce profiles having a very large number of features. In this context we can adopt a two-steps pre-filtering, e.g. using a fast univariate feature selection method to drop the less significant features and then using a second level multi-variate feature selection method to take into account feature interactions and refine the set of selected features (e.g. classical floating search or branch and bound methods)^85^. This framework can be integrated with *P-Net* by substituting its first two steps (data collection and construction of the patient similarity network) with appropriate procedure to construct the network with the different data. Then we have two options to accomplish the integration of the different networks: a) direct integration from the similarity matrices; b) integration after the application of a graph kernel (step 3 of the *P-Net* algorithm). In both cases we can apply suitable network-based data combination methods, such as simple averaging of the weights of the different networks, or weighted averaging according to the informativeness of each source of data^20,29,86^ or the SNF methods proposed in^83^. Considering the large range of possible clinical or omics data that can be used in this context^87^, and the different similarity measures and integration techniques that can be considered^88^, we believe that these problems need specific future studies and works that could expand the applicability and the effectiveness of the *P-Net* learning framework.

A limitation of the proposed approach is that *P-Net* requires potentially expensive feature selection algorithms to reduce the high dimensionality that usually characterizes genomic data, in order to remove irrelevent features that could add noise to the network of patients. For computational complexity reasons we applied simple univariate methods, but more refined multi-variate methods could lead to better results at the cost of a higher computational time complexity^89^.

## Supplementary information

Supplementary Information.
