# Supplementary material for: Network modeling of patients' biomolecular profiles for clinical phenotype/outcome prediction
Source: Sci Rep. 2020 Feb 27;10:3612. doi: 10.1038/s41598-020-60235-8 (PMC7046773; doi:10.1038/s41598-020-60235-8)
Supplement: Supplementary file 1 — Supplementary Information. [file 41598_2020_60235_MOESM1_ESM.pdf]

Network modeling of patients' biomolecular profiles for clinical phenotype/outcome prediction: Supplementary Information

Jessica Gliozzo[1, 2], Paolo Perlasca[1], Marco Mesiti[1], Elena Casiraghi[1], Viviana Vallacchi[3], Elisabetta Vergani[3], Marco Frasca[1], Giuliano Grossi[1], Alessandro Petrini[1], Matteo Re[1], Alberto Paccanaro[4 *] and Giorgio Valentini[1 *]

1 AnacletoLab - Dipartimento di Informatica, Università degli Studi di Milano, Milano, Italy

2 Department of Dermatology, Fondazione IRCCS Ca' Granda - Ospedale Maggiore Policlinico, Milan, Italy

3 Unit of Immunotherapy of Human Tumors, Fondazione Istituto di Ricovero e Cura a Carattere Scientifico (IRCCS) Istituto Nazionale dei Tumori di Milano, Milan, Italy

4 Centre for Systems and Synthetic Biology - Department of Computer Science, Royal Holloway, University of London, Egham, London, UK

* E-mail: alberto@cs.rhul.ac.uk, valentini@di.unimi.it

Contents

1 Datasets 4

2 Efficient implementation of *P-Net* 4

2.1 An efficient procedure to compute the "optimal" threshold 4

2.2 A double loo procedure for the network-based ranking of patients with *P-Net*. 6

2.3 A cross-validation-based implementation of *P-Net* 7

3 A non parametric test to validate patient ranking 8

4 Experimental set-up with Pancreatic cancer data 8

5 Experimental set-up for the analysis of Breast, Colorectal and Colon cancer patients 10

6 Summary of P-Net results 11

List of Tables

S1 **Summary of *P-Net* AUROC, AUPRC, Accuracy and F-score results.** For each dataset we provide the average value and the standard deviation (in brackets) achieved with different metrics. 11

S2 **P-Net methods applied to each dataset.** "RWK" stands for Random Walk Kernel followed by the number of steps. 14

S3 **Results with Breast, Colorectal and Colon cancer patients.** The results for each method are presented in the form **accuracy(sensitivity/specificity)** and the highest values for each dataset are in bold. For the TSVM only the accuracy values are provided. With *P-Net*, the Random Walk Kernel 1-step resulted the best choice for all the datasets; the differential normalized score achieved the highest accuracy in the Breast cancer dataset and the differential score the best results with the other datasets. 14

S4 **KEGG terms overrepresented in the hypergeometric test on Breast cancer dataset.** KEGG terms are linked to the corresponding pathway. The last column shows the reference supporting the association. 14

S5 **GO BP terms overrepresented in the hypergeometric test on Breast cancer dataset.** GO BP terms are linked to the corresponding entry in AmiGO 2 website. The last column shows the reference supporting the association. 15

S6 **KEGG terms overrepresented in the hypergeometric test on Colon cancer dataset.** KEGG terms are linked to the corresponding pathway. 15

S7 **GO BP terms overrepresented in the hypergeometric test on Colon cancer dataset.** GO BP terms are linked to the corresponding entry in AmiGO 2 website. 16
S8 **KEGG terms overrepresented in the hypergeometric test on Colorectal cancer dataset.** KEGG terms are linked to the corresponding pathway. 17
S9 **GO BP terms overrepresented in the hypergeometric test on Colorectal cancer dataset.** GO BP terms are linked to the corresponding entry in AmiGO 2 website. 17

List of Figures

S1 ***Optimize_thresh_by_loo***: a procedure to find the "optimal" filtering threshold τ. 5
S2 ***P-Net: double loo for network filtering and patient ranking*** 6
S3 ***P-Net* using cross-validation for network filtering and patient ranking** 7
S4 **One step of the Monte Carlo cross-validation procedure with P-Net.** 9
S5 **Breast cancer graph constructed by *P-Net*.** Square nodes represent "high risk", circles "low risk" of recurrence patients, while triangles represent unlabeled patients. The scores predicted by *P-Net* are represented in red (high risk) and white (low risk) with intermediate scores represented in pink. Two high risk subgroups of patients are outlined in red, while two subgroups of low risk patients are outlined in green. Unlabeled patients are predicted as low risk patients (outlined in gray). 12
S6 **IPA analysis**: top ten canonical pathways significantly enriched for high-risk recurrence in Colon-colorectal cancer (left) and breast cancer (right). Top: percentage of genes found in enriched pathways; bottom: $-\log(p - value)$ according to the Fisher exact test. 13

1 Datasets

The **Pancreatic cancer dataset** (downloadable from ArrayExpress Archive [1], accession number E-MEXP-2780) consists of microarray gene expression data from 30 patients that suffer from pancreatic ductal adenocarcinoma. Following [2], patients are split into the two groups *good prognosis* (GP; 15 patients) and *poor prognosis* (PP; 15 patients), depending on their time of survival. Patients are labelled as PP if their time of survival is below 17.5 months, otherwise the label is GP. Since the accurate prediction of PP patients is usually more interesting, they are treated as "positive patients" belonging to the group $V_C \subset V$ under study.

The data have been normalized using the Robust Multi-array Average (RMA) method [3]. We filtered out the genes with low expression and low variance, retaining only the probe sets with the highest mean expression for each gene (refer to [2] for more details about dataset pre-processing, filtering and labelling steps).

The gene expression datasets **Breast cancer** (GSE2990), **Colorectal cancer** (GSE17536), **Colon cancer** (GSE17538), are all downloadable from GEO, and have been normalized through RMA. The GSE2990 dataset is composed of 189 invasive breast cancer patients and 64 among them are unlabeled. The remaining ones are divided into the classes high and low risk of recurrence. The GSE17536 includes gene expression data for 177 colorectal cancer patients and the patients are divided into three groups "recurrence", "no recurrence" and "unlabeled". Finally, the GSE17538 dataset is made up of 213 colon cancer patients which are divided into the same three classes.

2 Efficient implementation of *P-Net*

The *P-Net* algorithm uses an optimized leave-one-out (LOO) procedure to evaluate both the generalization performance of the algorithm and to select the optimal threshold τ to filter out the least relevant edges of the network. In this section are detailed both the implementation of the efficient LOO procedure to filter the network, and the double LOO procedure to both estimate the generalization performance and to filter the network. Finally a cross-validated implementation of *P-Net* is provided. The R code implementing all these procedures is available from GitHub (`https://github.com/GliozzoJ/P-Net`).

2.1 An efficient procedure to compute the "optimal" threshold

Using the trick which allows us to run just once the *P-Net* algorithm for the loo, we can introduce the procedure *Optimize_thresh_by_loo* which exploits the efficient implementation of the internal loo to select the optimal network threshold τ (Fig. S1).

Figure S1: ***Optimize_thresh_by_loo***: a procedure to find the "optimal" filtering threshold τ.

`Input`:
- $\boldsymbol{K}$: a $n \times n$ kernel matrix of the graph
- $V_T \subseteq V$: the set of patients to be tested
- $V_C \subset V$: patients having the C phenotype
- S: a score function
- T: a set of thresholds to be tested

`begin`
01: $diag\ (\boldsymbol{K}) := 0$
02: $AUC^* := 0;\ t^* := 0$
03: `for each` $t \in T$
04: $\quad \boldsymbol{K}' := Filter\ (\boldsymbol{K}, t)$
05: $\quad$ `for each` $i \in V_T$
06: $\quad\quad s_i := S(i, \boldsymbol{K}'_i, V_C)$
07: $\quad$ `end for`
08: $\quad AUC := Compute\_AUC(\boldsymbol{s}, V_C)$
09: $\quad$ `if` $(AUC > AUC^*)$
10: $\quad\quad AUC^* := AUC$
11: $\quad\quad t^* := t$
12: $\quad$ `end if`
13: `end for`
`end.`

`Output`:
- t^*: the "optimal" threshold
- AUC^*: the AUC obtained by filtering the net with t^*

At first the diagonal of the kernel matrix $\boldsymbol{K}$ is set to zero (row 01). It is worthwhile to note that the input matrix in the algorithm can be or a kernel or a correlation matrix, but it has to be a symmetric matrix representing similarities between patients. In row 02 we initialize the optimal Area Under the Curve (AUC^*) and the optimal threshold t^* to zero. In the main *for loop* (rows $03-13$) a set of pre-selected thresholds $t \in T$ is tested to find the optimal threshold t^* which is the threshold that gives the best performances in term of AUC when it is used to filter the graph. We used a set of quantiles as pre-selected thresholds. In this loop, the algorithm computes a different filtered Kernel matrix $\boldsymbol{K}'$ at each iteration by choosing a different threshold t each time. The *Filter* function removes all the edges with a weight below the selected threshold. In the following inner *for loop* (rows $05-07$) the score function S is applied to the filtered kernel matrix to compute the score s_i of each node i. Only the nodes included in the subset V_T are tested. At row 08 the function *Compute_AUC* computes the AUC exploiting the vector of scores $\boldsymbol{s}$ obtained from the previous *for loop*. Then if the computed AUC is higher of the current maximal Area Under the Curve (AUC^*), the AUC^* and the corresponding threshold t^* are updated (rows $09-12$). At the end of the procedure the "optimal" threshold and the corresponding AUC are returned. The complexity of the algorithm *Optimize_thresh_by_loo* is $\mathcal{O}(n^2)$ if $|Q| << n$, otherwise the complexity is $\mathcal{O}(n^3)$. Indeed, within the *for each* loop the most expensive procedures are *Filter* (row 04) and the *for loop* at rows

05 – 07, both with complexity $\mathcal{O}(n^2)$.

2.2 A double loo procedure for the network-based ranking of patients with *P-Net*.

Fig. S2 shows the pseudo-code of the *P-Net* algorithm.

Figure S2: *P-Net: double loo for network filtering and patient ranking*

`Input`:
- $\boldsymbol{K}$: a $n \times n$ kernel matrix of the graph
- $V_C \subset V$: patients having the C phenotype
- S: a score function
- T: a set of thresholds to be tested

`begin`
01: `for` i `from` 1 `to` m
02: $t_i :=$ *Optimize_thresh_by_loo* $(\boldsymbol{K}, V \setminus \{i\}, V_C \setminus \{i\}, S, T)$
03: $\boldsymbol{K}'_i :=$ *Filter* $(\boldsymbol{K}_i, t_i)$
04: $k'_{ii} := 0$
05: $s_i := S(i, \boldsymbol{K}'_i, V_C)$
06: `end for`
`end`.

`Output`:
- the score vector $\boldsymbol{s}$
- the threshold vector $\boldsymbol{t}$

The double loo procedure is characterized by a main *for loop* (rows 01 – 06) necessary to obtain a score for each node i of the input matrix $\boldsymbol{K}$. So, the P-Net algorithm computes the "optimal" threshold t_i and the score s_i for each node applying the same steps node by node. In row 02 the function *Optimize_thresh_by_loo* implements the *internal loo* to select the "optimal" threshold for the ith node. In this loo the node i is left out and it is not considered in the computation. Then the threshold t_i is exploited to filter the ith row of the matrix $\boldsymbol{K}$. In the ith row all the elements below to t_i are set to zero and this is equivalent to cut the edges incident to the node i in the corresponding graph. In row 04 the element k'_{ii} is set to zero: this is needed to implement the *external loo* through the main for loop (rows 01 – 06). The last step (row 05) of the loop computes the score s_i using the filtered row K'_i. The outputs of this procedure are: a vector $\boldsymbol{s}$ which contains the scores for each node computed by external loo; a vector $\boldsymbol{t}$ which contains all the "optimal" thresholds selected for each sample by internal loo.

It is worthwhile to note that the *double loo procedure* seems to be computationally intensive but using the efficient implementation of the loo technique, the overall complexity of *P-Net* is $\mathcal{O}(n^3)$. Indeed, the *Optimize_thresh_by_loo* procedure has complexity $\mathcal{O}(n^2)$ when $|Q| << n$ is iterated n times.

2.3 A cross-validation-based implementation of *P-Net*

In this section we present another efficient implementation of *P-Net* by which the generalization performances are assessed through a classical k-fold cross-validation technique, while the selection of the "optimal" threshold is performed through the efficient implementation of the loo procedure.

The pseudocode of the cross-validated implementation of *P-Net* is presented in Fig S3. In row 01 the function *Split* divides randomly the vertices V in k folds. The main *for loop* evaluates the scores of the patients by external cross-validation (rows $02 - 08$). More precisely, inside the main *for loop*, the optimal threshold t_j is selected by internal loo through the *Optimize_thresh_by_loo* function. Then the matrix $\boldsymbol{K}$ is filtered using the optimal threshold for the fold V_j. Finally, in the second loop (rows $05 - 07$) the score for each node belonging to the test fold V_j is computed.

It is easy to see that the *hold-out* version of *P-Net*, by which data are split in training and test set, can be obtained from the cross-validation version of Fig. S3. Indeed we can substitute the *Split* function at row 01 of Fig. S3 with a simpler splitting of the vertices V in training and test set. Then the outer *for loop* can be eliminated: it is sufficient a single iteration of the *for loop* at rows $02 - 08$, and finally V_j can be substituted with the test set.

Figure S3: *P-Net* using cross-validation for network filtering and patient ranking

`Input`:
- $\boldsymbol{K}$: a $n \times n$ kernel matrix of the graph
- $V_C \subset V$: patients having the C phenotype
- k: number of folds for the cross-validation
- S: a score function
- T: a set of thresholds to be tested

`begin`
01: $\{V_1, \ldots, V_k\}$:= *Split* (V, k)
02:   `for` j `from` 1 `to` k
03:     t_j := *Optimize_thresh_by_loo* $(\boldsymbol{K}, V \setminus V_j, V_C \setminus V_j, S, T)$
04:     $\boldsymbol{K}'$:= *Filter* $(\boldsymbol{K}, t_j)$
05:     `for each` $i \in V_j$
06:       $s_i := S(i, \boldsymbol{K}'_i, V_C \setminus V_j)$
07:     `end for`
08:   `end for`
`end`.

`Output`:
- the score vector $\boldsymbol{s}$
- the threshold vector $\boldsymbol{t}$

3 A non parametric test to validate patient ranking

To evaluate whether the ranking of the patients has been obtained by chance, we can apply a classical non parametric test based on random shuffling of patients' labels. Having a kernel matrix $\boldsymbol{K}$ we can apply multiple random shufflings (e.g. k times) of the set of the labels of patients. Then we apply *P-Net* with both the "unshuffled" and with the shuffled set of labels, and we compute the AUC and the scores for each patient for both the unshuffled and shuffled version of the labels. More precisely at each iteration i of the shuffling we compare the AUC obtained by *P-Net* with the "true labels" (AUC_T) with respect to the AUC obtained with the "shuffled labels" ($AUC_S^{(i)}$). We can obtain the following *p-value* of the ranking, by counting how many times the AUC computed with the shuffled labels is larger or equal than the AUC computed with the true labels:

$$\text{p-value} = \frac{1}{k}\sum_{i=1}^{k}\{AUC_T \leq AUC_S^{(i)}\} \tag{1}$$

where the operator $\{P\}$ returns 1 if the predicate P is true, otherwise 0.

4 Experimental set-up with Pancreatic cancer data

The generalization performances have been evaluated through a Monte Carlo cross-validation technique (MCCV), i.e. a hold-out procedure repeated 1000 times (see Fig. S4).

Figure S4: One step of the Monte Carlo cross-validation procedure with P-Net.

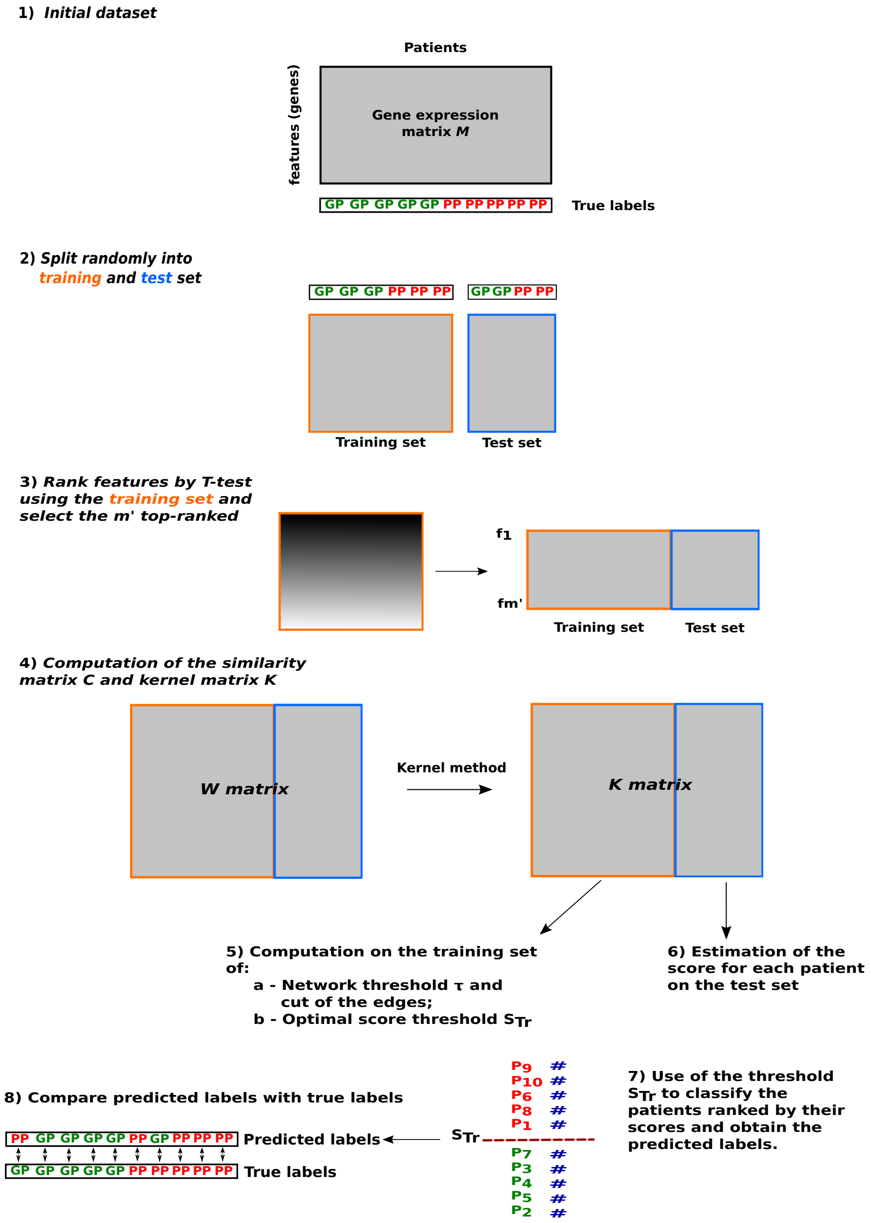

The main steps of the *P-Net* MCCV can be summarized as follows:

1. The data are randomly split into training set and test set using a stratified approach to obtain a balanced representation of the two considered classes (GP and PP) into the sets.
2. Student's t-test is applied to the training set data only, and the 1000 genes with the smallest p-value are selected.

3. P-Net is executed using only the features selected in the precedent step. The optimization of the edge threshold τ is carried out by internal leave-one-out on the training set data and the estimation of the scores is performed on the test set.

4. P-Net assigns a score to each patient, and the "optimal" score threshold to separate PP from GP patients is assessed on the training set.

5. The above score threshold is then used to classify each patient of the test set: patients having a score above the threshold are classified as PP, otherwise as GP.

6. Predicted and "true" labels are compared to calculate the accuracy as fraction of correctly predicted patients.

After 1000 rounds of the above procedure, the average accuracy across the rounds is computed.

5 Experimental set-up for the analysis of Breast, Colorectal and Colon cancer patients

Park et al. compared their method with three supervised classification algorithms (Support Vector Machine, Naïve Bayes, Random Forest) and with the semi-supervised version of SVM (TSVM). Following the same approach of [4], the performances have been evaluated through a 10-fold cross validation procedure repeated 15 times on each dataset, using as metrics the accuracy, sensitivity, specificity and AUC averaged across all the repetitions.

Park et al. used PPI information to filter out genes having no known interactions and to identify the most informative pairs of genes [4]. Even if PPI interactions can be useful to identify functional relationships between genes, to avoid filtering genes only on the basis of their known interactions with other genes, we simply applied a t-test to select informative genes to be used with *P-Net*. The following steps summarize the set-up we used for a single repetition of the 10-fold cross validation with *P-Net*:

1. We applied the Student's t-test on each independent probe using all the gene expression data from the labeled patients of the dataset. Then we retained all the probes having a Bonferroni corrected p-value < 0.05.

2. We constructed the similarity matrix $\boldsymbol{W}$ using Pearson correlation between all the available patients (labeled and unlabeled ones).

3. Then we applied a Random Walk Kernel on the resulting similarity matrix $\boldsymbol{W}$.

4. The resulting Kernel matrix is filtered to remove all the edges having a weight below the selected threshold τ.

5. The performances on the test set are evaluated using accuracy, sensitivity, specificity and AUC averaged across the repetitions of the cross validation. To find the *optimal score threshold* in an unbiased way, we computed the

scores on the training set and we tested an arbitrary vector of quantiles to classify the patients and to compute the corresponding accuracy. The *optimal score threshold* is equal to the quantile that returns the best accuracy on the training set.

6. A *score function* is then used to compute the scores on the test set and the *optimal score threshold* is employed to classify the patients of the test set according to their scores.

7. Finally, we compared our predictions with the labels and we computed all the metrics for a single repetition of the 10-fold cross validation

8. The procedure is repeated 15 times and all the metrics are averaged across the repetitions to get the final results.

6 Summary of P-Net results

Table S1 shows the average AUROC, AUPRC, Accuracy and F-score and their respective standard deviation obtained using *P-Net* on the considered datasets. Results obtained with Breast, Colorectal and Colon data sets are significantly more stable than those obtained with Pancreatic cancer data: this is not surprising since the cardinality of the first three datasets is significantly larger than that of Pancreatic cancer. For this latter data set we reported also the results for different sizes of the training set, according to the set-up proposed in [2]: by reducing the test size, as expected, the standard deviation significantly increases, since the Monte-Carlo cross-validation, with a very small data set tends to behave like a leave-one-out procedure, that is known to be characterized by a large variance.

Table S1: Summary of *P-Net* AUROC, AUPRC, Accuracy and F-score results. For each dataset we provide the average value and the standard deviation (in brackets) achieved with different metrics.

Dataset	AUROC		AUPRC		Accuracy		F-score	
Breast	0.915	(0.019)	0.848	(0.036)	0.841	(0.026)	0.793	(0.034)
Colorectal	0.784	(0.021)	0.499	(0.049)	0.790	(0.017)	0.352	(0.053)
Colon	0.744	(0.018)	0.516	(0.045)	0.764	(0.012)	0.360	(0.043)
Pancreatic (Training set size 0.5)	0.688	(0.149)	0.662	(0.128)	0.642	(0.111)	0.639	(0.137)
Pancreatic (Training set size 0.6)	0.704	(0.160)	0.679	(0.139)	0.649	(0.122)	0.644	(0.144)
Pancreatic (Training set size 0.7)	0.722	(0.175)	0.701	(0.155)	0.660	(0.130)	0.658	(0.146)
Pancreatic (Training set size 0.8)	0.753	(0.220)	0.755	(0.193)	0.680	(0.185)	0.697	(0.186)
Pancreatic (Training set size 0.9)	0.781	(0.404)	0.833	(0.297)	0.781	(0.404)	0.786	(0.401)

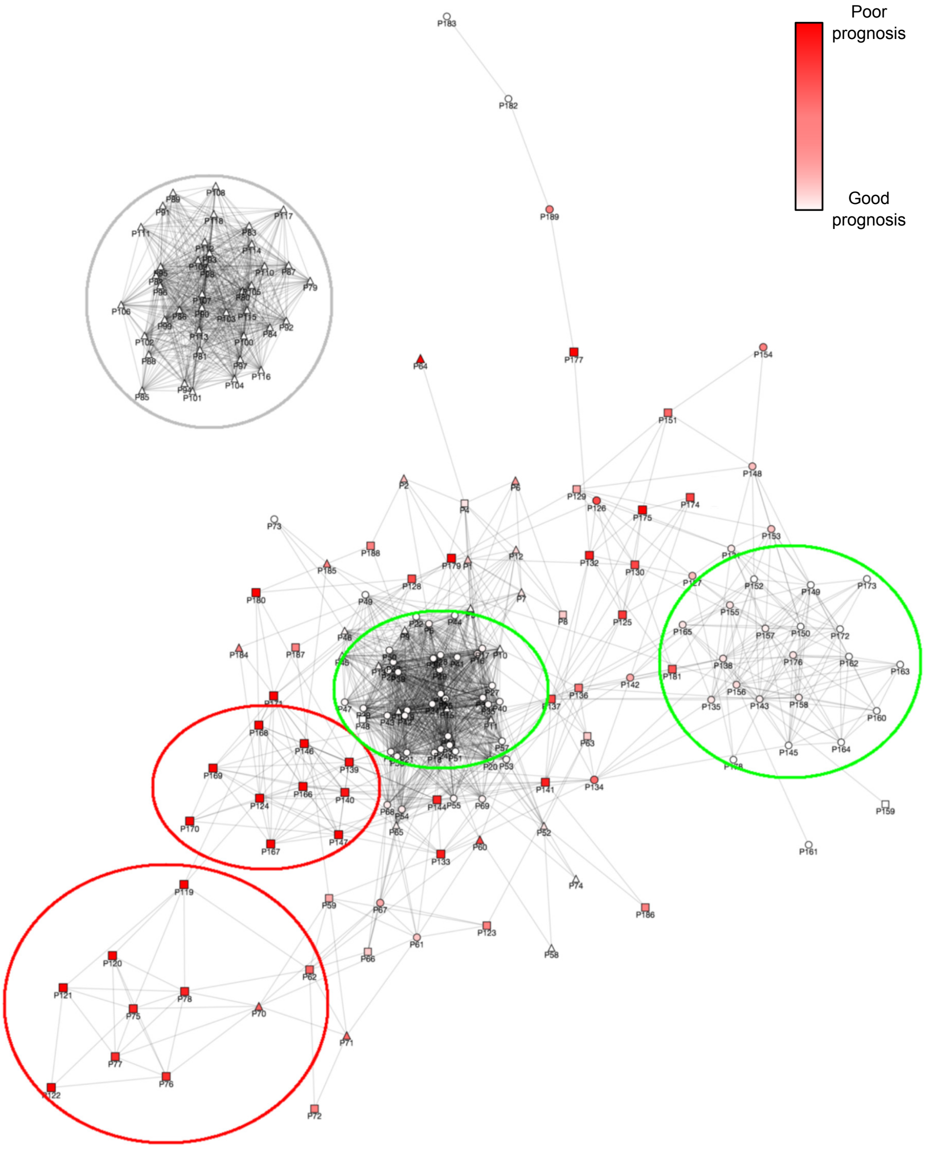

Figure S5: Breast cancer graph constructed by *P-Net*. Square nodes represent "high risk", circles "low risk" of recurrence patients, while triangles represent unlabeled patients. The scores predicted by *P-Net* are represented in red (high risk) and white (low risk) with intermediate scores represented in pink. Two high risk subgroups of patients are outlined in red, while two subgroups of low risk patients are outlined in green. Unlabeled patients are predicted as low risk patients (outlined in gray).

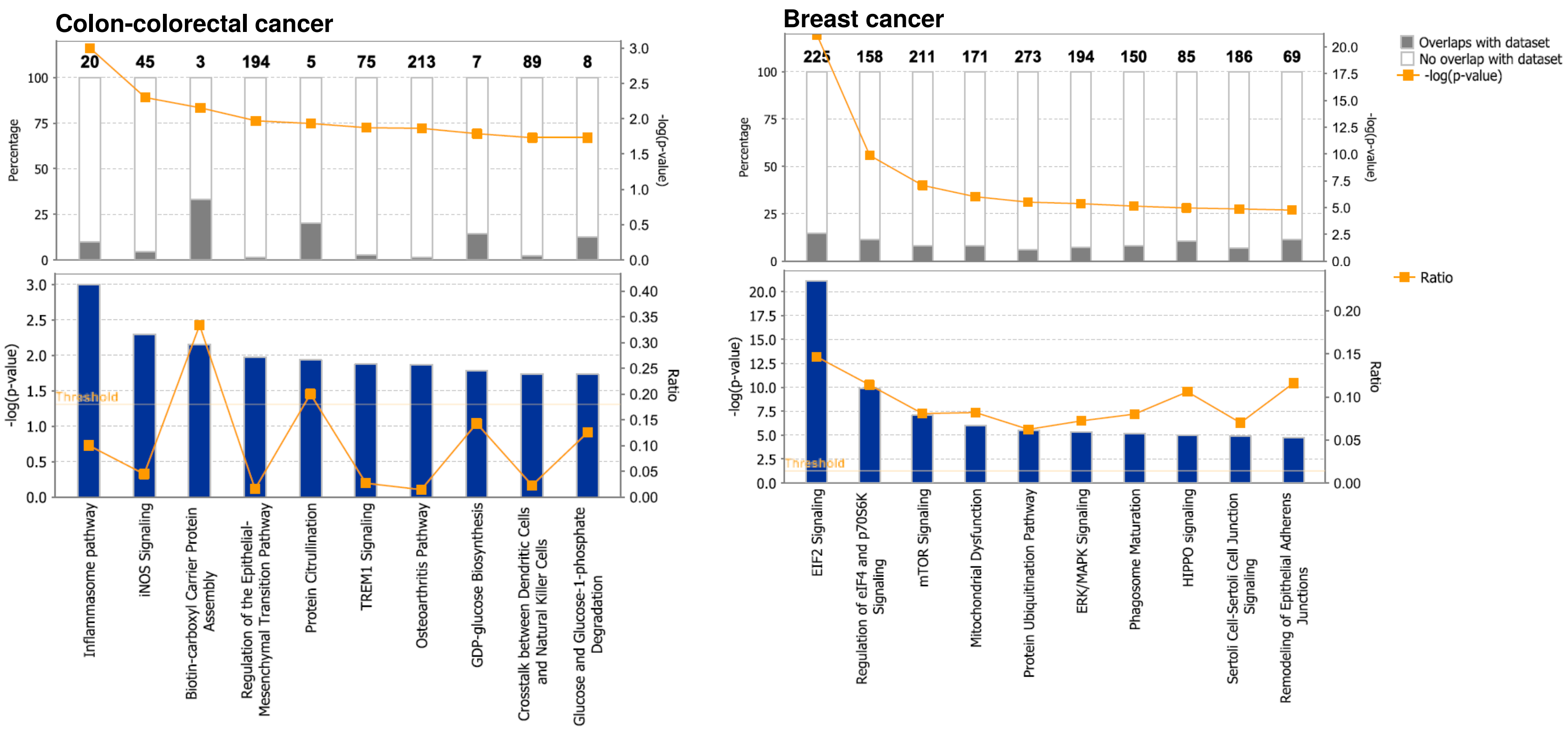

Figure S6: IPA analysis: top ten canonical pathways significantly enriched for high-risk recurrence in Colon-colorectal cancer (left) and breast cancer (right). Top: percentage of genes found in enriched pathways; bottom: $-\log(p-value)$ according to the Fisher exact test.

Table S2: P-Net methods applied to each dataset. "RWK" stands for Random Walk Kernel followed by the number of steps.

Dataset	Kernel	Score Function
Pancreatic	RWK 8-steps	Nearest Neighbour
Breast	RWK 1-step	Differential Normalized
Colorectal	RWK 1-step	Differential
Colon	RWK 1-step	Differential

Table S3: Results with Breast, Colorectal and Colon cancer patients. The results for each method are presented in the form **accuracy(sensitivity/specificity)** and the highest values for each dataset are in bold. For the TSVM only the accuracy values are provided. With *P-Net*, the Random Walk Kernel 1-step resulted the best choice for all the datasets; the differential normalized score achieved the highest accuracy in the Breast cancer dataset and the differential score the best results with the other datasets.

Method	Breast (GSE2990)	Colorectal (GSE17536)	Colon (GSE17538)
P-Net	**0.841** (0.778/**0.884**)	0.790 (0.233/**0.974**)	0.764 (0.253/0.954)
Park Method	0.725 (0.617/0.795)	**0.807** (0.485/0.906)	0.756 (0.163/**0.977**)
TSVM	0.543 (-/-)	0.752 (-/-)	0.728 (-/-)
SVM	0.528 (0.671/0.306)	0.772 (0.889/0.389)	**0.796** (0.917/0.469)
Naïve Bayes	0.592 (0.605/0.571)	0.759 (0.844/0.500)	0.707 (0.826/0.388)
Random Forest	0.664 (**0.921**/0.265)	0.752 (**0.963**/0.111)	0.713 (**0.955**/0.061)

Table S4: KEGG terms overrepresented in the hypergeometric test on Breast cancer dataset. KEGG terms are linked to the corresponding pathway. The last column shows the reference supporting the association.

KEGG IDs	p-value	Term	References
03010	0.000	Ribosome	[5, 6]
03050	0.000	Proteasome	[7, 8]
04141	0.003	Protein processing in endoplasmic reticulum	[9, 10]
00190	0.003	Oxidative phosphorylation	[11]
03040	0.009	Spliceosome	[12]
04510	0.013	Focal adhesion	[13, 14]
00480	0.025	Glutathione metabolism	[15, 16]
00970	0.028	Aminoacyl-tRNA biosynthesis	[17]
03013	0.029	RNA transport	[18]
00270	0.048	Cysteine and methionine metabolism	[19]

Table S5: GO BP terms overrepresented in the hypergeometric test on Breast cancer dataset. GO BP terms are linked to the corresponding entry in AmiGO 2 website. The last column shows the reference supporting the association.

GO IDs	p-value	Term	References
GO:0002479	0.000	antigen processing and presentation of exogenous peptide antigen via MHC class I, TAP-dependent	[20]
GO:0090263	0.000	positive regulation of canonical Wnt signaling pathway	[21]
GO:0044770	0.000	cell cycle phase transition	[22]
GO:0038061	0.000	NIK/NF-kappaB signaling	[23]
GO:0006413	0.000	translational initiation	[24]
GO:0000375	0.000	RNA splicing, via transesterification reactions	[12]
GO:0033209	0.000	tumor necrosis factor-mediated signaling pathway	[25]
GO:0035026	0.001	leading edge cell differentiation	[26, 27]
GO:0097435	0.001	supramolecular fiber organization	[28]
GO:0097190	0.001	apoptotic signaling pathway	[29]
GO:0030511	0.001	positive regulation of transforming growth factor beta receptor signaling pathway	[30]
GO:2000819	0.003	regulation of nucleotide-excision repair	[31]
GO:0072331	0.005	signal transduction by p53 class mediator	[32]
GO:0098869	0.006	cellular oxidant detoxification	[33]
GO:0006338	0.006	chromatin remodeling	[34]
GO:0048863	0.009	stem cell differentiation	[35]

Table S6: KEGG terms overrepresented in the hypergeometric test on Colon cancer dataset. KEGG terms are linked to the corresponding pathway.

KEGG IDs	p-value	Term	References
00061	0.002	Fatty acid biosynthesis	[36]
00640	0.012	Propanoate metabolism	[37]
00620	0.015	Pyruvate metabolism	[37]
04920	0.025	Adipocytokine signaling pathway	[38, 39]
05160	0.049	Hepatitis C	[40]

Table S7: GO BP terms overrepresented in the hypergeometric test on Colon cancer dataset. GO BP terms are linked to the corresponding entry in AmiGO 2 website.

GO IDs	p-value	Term	References
GO:2001295	0.001	malonyl-CoA biosynthetic process	[41]
GO:0031999	0.003	negative regulation of fatty acid beta-oxidation	[42]
GO:0038110	0.003	interleukin-2-mediated signaling pathway	[43]
GO:0038113	0.003	interleukin-9-mediated signaling pathway	[44, 45]
GO:0071104	0.004	response to interleukin-9	[44]
GO:0070669	0.005	response to interleukin-2	[46]
GO:0006853	0.006	carnitine shuttle	[47]
GO:0006768	0.006	biotin metabolic process	[48, 49]
GO:0035723	0.008	interleukin-15-mediated signaling pathway	[50]
GO:0070672	0.008	response to interleukin-15	[50, 51]
GO:0006346	0.010	methylation-dependent chromatin silencing	[52, 53]
GO:2000738	0.010	positive regulation of stem cell differentiation	[54]
GO:0010884	0.011	positive regulation of lipid storage	[55]
GO:1903672	0.011	positive regulation of sprouting angiogenesis	[56]
GO:0045922	0.013	negative regulation of fatty acid metabolic process	[42]
GO:0060334	0.014	regulation of interferon-gamma-mediated signaling pathway	[57]
GO:0046320	0.016	regulation of fatty acid oxidation	[58]
GO:0006084	0.019	acetyl-CoA metabolic process	[59]
GO:0038111	0.019	interleukin-7-mediated signaling pathway	[60, 61]
GO:0097009	0.019	energy homeostasis	[62]
GO:0098760	0.020	response to interleukin-7	[61]
GO:0045540	0.024	regulation of cholesterol biosynthetic process	[63]
GO:0060338	0.025	regulation of type I interferon-mediated signaling pathway	[64]
GO:0035384	0.028	thioester biosynthetic process	[65]
GO:0050994	0.028	regulation of lipid catabolic process	[66]
GO:0035722	0.031	interleukin-12-mediated signaling pathway	[67]
GO:0070671	0.031	response to interleukin-12	[67]
GO:0016126	0.041	sterol biosynthetic process	[68]
GO:0051304	0.042	chromosome separation	[69]
GO:0038094	0.047	Fc-gamma receptor signaling pathway	[70]
GO:0046620	0.047	regulation of organ growth	[71]
GO:0071357	0.048	cellular response to type I interferon	[64]
GO:0015908	0.050	fatty acid transport	[72]

Table S8: KEGG terms overrepresented in the hypergeometric test on Colorectal cancer dataset. KEGG terms are linked to the corresponding pathway.

KEGG IDs	p-value	Term	References
05130	0.006	Pathogenic Escherichia coli infection	[73, 74]
00533	0.037	Glycosaminoglycan biosynthesis - keratan sulfate	[75, 76]

Table S9: GO BP terms overrepresented in the hypergeometric test on Colorectal cancer dataset. GO BP terms are linked to the corresponding entry in AmiGO 2 website.

GO IDs	p-value	Term	References
GO:0060294	0.001	cilium movement involved in cell motility	[77]
GO:0098581	0.001	detection of external biotic stimulus	[78]
GO:1904886	0.002	beta-catenin destruction complex disassembly	[79]
GO:0035411	0.002	catenin import into nucleus	[80]
GO:1904793	0.003	regulation of euchromatin binding	[81]
GO:1900170	0.003	negative regulation of glucocorticoid mediated signaling pathway	[82]
GO:0001539	0.003	cilium or flagellum-dependent cell motility	[77]
GO:0070434	0.003	positive regulation of nucleotide-binding oligomerization domain containing 2 signaling pathway	[83]
GO:0045429	0.005	positive regulation of nitric oxide biosynthetic process	[84]
GO:0002537	0.006	nitric oxide production involved in inflammatory response	[84, 85]
GO:0050702	0.006	interleukin-1 beta secretion	[86]
GO:0045976	0.006	negative regulation of mitotic cell cycle, embryonic	[87]
GO:0036413	0.006	histone H3-R26 citrullination	[88]
GO:1901895	0.008	negative regulation of calcium-transporting ATPase activity	[89]
GO:0010909	0.008	positive regulation of heparan sulfate proteoglycan biosynthetic process	[90]
GO:0002357	0.011	defense response to tumor cell	[91]
GO:0045950	0.011	negative regulation of mitotic recombination	[87]
GO:0002322	0.014	B cell proliferation involved in immune response	[92]
GO:1904885	0.014	beta-catenin destruction complex assembly	[93]
GO:0007197	0.014	adenylate cyclase-inhibiting G-protein coupled acetylcholine receptor signaling pathway	[94]
GO:1900227	0.014	positive regulation of NLRP3 inflammasome complex assembly	[95]
GO:0070269	0.022	pyroptosis	[96]
GO:0061448	0.025	connective tissue development	[97]
GO:0051091	0.028	positive regulation of DNA binding transcription factor activity	[98]
GO:0051770	0.039	positive regulation of nitric-oxide synthase biosynthetic process	[84]
GO:0007252	0.041	I-kappaB phosphorylation	[99]
GO:0001906	0.049	cell killing	[100]

References

[1] H. Parkinson, M. Kapushesky, M. Shojatalab, N. Abeygunawardena, R. Coulson, A. Farne, E. Holloway, N. Kolesnykov, P. Lilja, M. Lukk, *et al.*, "ArrayExpress—a public database of microarray experiments and gene expression profiles," *Nucleic Acids Research*, vol. 35, no. suppl 1, pp. D747–D750, 2007.

[2] C. Winter *et al.*, "Google Goes Cancer: Improving Outcome Prediction for Cancer Patients by Network-Based Ranking of Marker Genes," *PLoS Computational Biology*, vol. 8, no. 5, 2012.

[3] B. Bolstad, R. Irizarry, M. Astrand, and T. Speed, "A comparison of normalization methods for high density oligonucleotide array data based on variance and bias," *Bioinformatics*, vol. 19, no. 2, pp. 185–193, 2003.

[4] C. Park, J. Ahn, H. Kim, and S. Park, "Integrative gene network construction to analyze cancer recurrence using semi-supervised learning," *PLOS ONE*, vol. 9, pp. 1–9, 01 2014.

[5] S. O. Sulima, I. J. Hofman, K. De Keersmaecker, and J. D. Dinman, "How ribosomes translate cancer," *Cancer Discovery*, vol. 7, no. 10, pp. 1069–1087, 2017.

[6] A. Bastide and A. David, "The ribosome,(slow) beating heart of cancer (stem) cell," *Oncogenesis*, vol. 7, no. 4, p. 34, 2018.

[7] F. Cardoso, J. S. Ross, M. J. Piccart, C. Sotiriou, and V. Durbecq, "Targeting the ubiquitin—proteasome pathway in breast cancer," *Clinical Breast Cancer*, vol. 5, no. 2, pp. 148 – 157, 2004.

[8] M. S. F. Roeten, J. Cloos, and G. Jansen, "Positioning of proteasome inhibitors in therapy of solid malignancies," *Cancer Chemotherapy and Pharmacology*, vol. 81, pp. 227–243, Feb 2018.

[9] C.-c. Han and F.-s. Wan, "New insights into the role of endoplasmic reticulum stress in breast cancer metastasis," *Journal of breast cancer*, vol. 21, no. 4, pp. 354–362, 2018.

[10] L. Sisinni, M. Pietrafesa, S. Lepore, F. Maddalena, V. Condelli, F. Esposito, and M. Landriscina, "Endoplasmic reticulum stress and unfolded protein response in breast cancer: The balance between apoptosis and autophagy and its role in drug resistance," *International Journal of Molecular Sciences*, vol. 20, no. 4, 2019.

[11] A. Mencalha, V. J. Victorino, R. Cecchini, and C. Panis, "Mapping oxidative changes in breast cancer: understanding the basic to reach the clinics," *Anticancer research*, vol. 34, no. 3, pp. 1127–1140, 2014.

[12] A. Read and R. Natrajan, "Splicing dysregulation as a driver of breast cancer," *Endocrine-related cancer*, vol. 25, no. 9, pp. R467–R478, 2018.

[13] X. Zhao and J.-L. Guan, "Focal adhesion kinase and its signaling pathways in cell migration and angiogenesis," *Advanced drug delivery reviews*, vol. 63, no. 8, pp. 610–615, 2011.

[14] A. Iuliano, A. Occhipinti, C. Angelini, I. De Feis, and P. Liò, "Combining pathway identification and breast cancer survival prediction via screening-network methods," *Frontiers in Genetics*, vol. 9, p. 206, 2018.

[15] A. Bansal and M. C. Simon, "Glutathione metabolism in cancer progression and treatment resistance," *The Journal of Cell Biology*, vol. 217, no. 7, pp. 2291–2298, 2018.

[16] T. Miran, A. T. J. Vogg, N. Drude, F. M. Mottaghy, and A. Morgenroth, "Modulation of glutathione promotes apoptosis in triple-negative breast cancer cells," *The FASEB Journal*, vol. 32, no. 5, pp. 2803–2813, 2018. PMID: 29301945.

[17] S.-q. Huang, B. Sun, Z.-p. Xiong, Y. Shu, H.-h. Zhou, W. Zhang, J. Xiong, and Q. Li, "The dysregulation of trnas and trna derivatives in cancer," *Journal of Experimental & Clinical Cancer Research*, vol. 37, no. 1, p. 101, 2018.

[18] B. Culjkovic-Kraljacic and K. L. Borden, "Aiding and abetting cancer: mrna export and the nuclear pore," *Trends in cell biology*, vol. 23, no. 7, pp. 328–335, 2013.

[19] P. Cavuoto and M. F. Fenech, "A review of methionine dependency and the role of methionine restriction in cancer growth control and life-span extension," *Cancer treatment reviews*, vol. 38, no. 6, pp. 726–736, 2012.

[20] P. Leone, E.-C. Shin, F. Perosa, A. Vacca, F. Dammacco, and V. Racanelli, "MHC Class I Antigen Processing and Presenting Machinery: Organization, Function, and Defects in Tumor Cells," *JNCI: Journal of the National Cancer Institute*, vol. 105, pp. 1172–1187, 07 2013.

[21] M. M. Kazi, T. I. Trivedi, T. P. Kobawala, N. R. Ghosh, *et al.*, "The potential of wnt signaling pathway in cancer: A focus on breast cancer," *Cancer Translational Medicine*, vol. 2, no. 2, p. 55, 2016.

[22] J. J. Bower, L. D. Vance, M. Psioda, S. L. Smith-Roe, D. A. Simpson, J. G. Ibrahim, K. A. Hoadley, C. M. Perou, and W. K. Kaufmann, "Patterns of cell cycle checkpoint deregulation associated with intrinsic molecular subtypes of human breast cancer cells," *NPJ Breast Cancer*, vol. 3, no. 1, p. 9, 2017.

[23] L. Xia, S. Tan, Y. Zhou, J. Lin, H. Wang, L. Oyang, Y. Tian, L. Liu, M. Su, H. Wang, *et al.*, "Role of the nfκb-signaling pathway in cancer," *OncoTargets and therapy*, vol. 11, p. 2063, 2018.

[24] A. Akcakanat, D. S. Hong, and F. Meric-Bernstam, "Targeting translation initiation in breast cancer," *Translation*, vol. 2, no. 1, p. e28968, 2014.

[25] I. Martínez-Reza, L. Díaz, and R. García-Becerra, "Preclinical and clinical aspects of tnf-α and its receptors tnfr1 and tnfr2 in breast cancer," *Journal of biomedical science*, vol. 24, no. 1, p. 90, 2017.

[26] R. W. Tilghman, J. K. Slack-Davis, N. Sergina, K. H. Martin, M. Iwanicki, E. D. Hershey, H. E. Beggs, L. F. Reichardt, and J. T. Parsons, "Focal adhesion kinase is required for the spatial organization of the leading edge

in migrating cells," *Journal of Cell Science*, vol. 118, no. 12, pp. 2613–2623, 2005.

[27] K. Bijian, C. Lougheed, J. Su, B. Xu, H. Yu, J. Wu, K. Riccio, and M. Alaoui-Jamali, "Targeting focal adhesion turnover in invasive breast cancer cells by the purine derivative reversine," *British journal of cancer*, vol. 109, no. 11, p. 2810, 2013.

[28] S. Tavares, A. F. Vieira, A. V. Taubenberger, M. Araújo, N. P. Martins, C. Brás-Pereira, A. Polónia, M. Herbig, C. Barreto, O. Otto, *et al.*, "Actin stress fiber organization promotes cell stiffening and proliferation of pre-invasive breast cancer cells," *Nature communications*, vol. 8, p. 15237, 2017.

[29] J. Plati, O. Bucur, and R. Khosravi-Far, "Dysregulation of apoptotic signaling in cancer: molecular mechanisms and therapeutic opportunities," *Journal of cellular biochemistry*, vol. 104, no. 4, pp. 1124–1149, 2008.

[30] J. M. Zarzynska, "Two faces of tgf-beta1 in breast cancer," *Mediators of inflammation*, vol. 2014, 2014.

[31] J. J. Latimer, J. M. Johnson, C. M. Kelly, T. D. Miles, K. A. Beaudry-Rodgers, N. A. Lalanne, V. G. Vogel, A. Kanbour-Shakir, J. L. Kelley, R. R. Johnson, *et al.*, "Nucleotide excision repair deficiency is intrinsic in sporadic stage i breast cancer," *Proceedings of the National Academy of Sciences*, vol. 107, no. 50, pp. 21725–21730, 2010.

[32] D. E. Moulder, D. Hatoum, E. Tay, Y. Lin, and E. M. McGowan, "The roles of p53 in mitochondrial dynamics and cancer metabolism: The pendulum between survival and death in breast cancer?," *Cancers*, vol. 10, no. 6, 2018.

[33] A. R. Nourazarian, P. Kangari, and A. Salmaninejad, "Roles of oxidative stress in the development and progression of breast cancer," *Asian Pac J Cancer Prev*, vol. 15, no. 12, pp. 4745–51, 2014.

[34] L. Manelyte, "Chromatin remodelers, their implication in cancer and therapeutic potential," *Journal of Rare Diseases Research & Treatment*, vol. 2, no. 3, pp. 34–40, 2017.

[35] W. C. Sin and C. L. Lim, "Breast cancer stem cells—from origins to targeted therapy," *Stem cell investigation*, vol. 4, 2017.

[36] Y. Y. Zaytseva, P. G. Rychahou, A.-T. Le, T. L. Scott, R. M. Flight, J. T. Kim, J. Harris, J. Liu, C. Wang, A. J. Morris, *et al.*, "Preclinical evaluation of novel fatty acid synthase inhibitors in primary colorectal cancer cells and a patient-derived xenograft model of colorectal cancer," *Oncotarget*, vol. 9, no. 37, p. 24787, 2018.

[37] T. Fan, G. Sun, X. Sun, L. Zhao, R. Zhong, and Y. Peng, "Tumor energy metabolism and potential of 3-bromopyruvate as an inhibitor of aerobic glycolysis: Implications in tumor treatment," *Cancers*, vol. 11, no. 3, 2019.

[38] D. Housa, J. Housova, Z. Vernerova, and M. Haluzik, "Adipocytokines and cancer.," *Physiological research*, vol. 55, no. 3, 2006.

[39] J. Terzić, S. Grivennikov, E. Karin, and M. Karin, "Inflammation and colon cancer," *Gastroenterology*, vol. 138, no. 6, pp. 2101 – 2114.e5, 2010. Colon Cancer: An Update and Future Directions.

[40] R. D. Allison, X. Tong, A. C. Moorman, K. N. Ly, L. Rupp, F. Xu, S. C. Gordon, S. D. Holmberg, C. H. C. S. C. Investigators, *et al.*, "Increased incidence of cancer and cancer-related mortality among persons with chronic hepatitis c infection, 2006–2010," *Journal of hepatology*, vol. 63, no. 4, pp. 822–828, 2015.

[41] D. Stepanova, G. Semenova, Y.-M. Kuo, A. J. Andrews, S. Ammoun, C. Hanemann, and J. Chernoff, "An essential role for the tumor-suppressor merlin in regulating fatty acid synthesis," *Cancer Research*, vol. CAN-16-2834, 2017.

[42] C. Kuo and D. Ann, "When fats commit crimes: fatty acid metabolism, cancer stemness and therapeutic resistance.," *Cancer Commun*, vol. 38, 2018.

[43] A. Chang, "Perspectives on interleukin-2 immunotherapy for colon cancer," *GASTROENTEROLOGY*, vol. 107, pp. 1890–1892, 1994.

[44] J. Wang, M. Sun, H. Zhao, Y. Huang, D. Li, D. Mao, Z. Zhang, X. Zhu, X. Dong, and X. Zhao, "Il-9 exerts antitumor effects in colon cancer and transforms the tumor microenvironment in vivo," *Technology in cancer research & treatment*, vol. 18, p. 1533033819857737, January 2019.

[45] Y. Lu, B. Hong, H. Li, Y. Zheng, M. Zhang, S. Wang, J. Qian, and Q. Yi, "Tumor-specific il-9–producing cd8+ tc9 cells are superior effector than type-i cytotoxic tc1 cells for adoptive immunotherapy of cancers," *Proceedings of the National Academy of Sciences*, vol. 111, no. 6, pp. 2265–2270, 2014.

[46] S. A. Rosenberg, M. T. Lotze, L. M. Muul, A. E. Chang, F. P. Avis, S. Leitman, W. M. Linehan, C. N. Robertson, R. E. Lee, J. T. Rubin, C. A. Seipp, C. G. Simpson, and D. E. White, "A progress report on the treatment of 157 patients with advanced cancer using lymphokine-activated killer cells and interleukin-2 or high-dose interleukin-2 alone," *New England Journal of Medicine*, vol. 316, no. 15, pp. 889–897, 1987. PMID: 3493432.

[47] M. Melone, A. Valentino, S. Margarucci, U. Galderisi, A. Giordano, and G. Peluso, "The carnitine system and cancer metabolic plasticity," *Cell Death Dis.*, vol. 9, p. 228, 2018.

[48] H. A. e. a. Rowland I, Gibson G, "Gut microbiota functions: metabolism of nutrients and other food components," *Eur J Nutr.*, vol. 57, pp. 1–24, 2018.

[49] J. R. e. a. Marchesi, "The gut microbiota and host health: a new clinical frontier," *Gut. 2016 Feb; 65(2): 330–339.*, vol. 65, pp. 330–339, 2016.

[50] J. C. Steel, T. A. Waldmann, and J. C. Morris, "Interleukin-15 biology and its therapeutic implications in cancer," *Trends in Pharmacological Sciences*, vol. 33, no. 1, pp. 35 – 41, 2012.

[51] H. Kuniyasu, H. Ohmori, T. Sasaki, T. Sasahira, K. Yoshida, Y. Kitadai, and I. J. Fidler, "Production of interleukin 15 by human colon cancer cells is associated with induction of mucosal hyperplasia, angiogenesis, and metastasis," *Clinical Cancer Research*, vol. 9, no. 13, pp. 4802–4810, 2003.

[52] A. El-Osta, "Dnmt cooperativity—the developing links between methylation, chromatin structure and cancer," *BioEssays*, vol. 25, no. 11, pp. 1071–1084, 2003.

[53] Y. Liang, C. Zhang, and D.-Q. Dai, "Identification of differentially expressed genes regulated by methylation in colon cancer based on bioinformatics analysis," *World journal of gastroenterology*, vol. 25, no. 26, p. 3392, 2019.

[54] M. e. a. Stoian, "Stem cells and colorectal carcinogenesis," *Journal of medicine and life*, vol. 9, pp. 6–11, 2016.

[55] J. e. a. Long, "Lipid metabolism and carcinogenesis, cancer development.," *American journal of cancer research*, vol. 8, pp. 778–791, 2018.

[56] I. Zuazo-Gaztelu and O. Casanovas, "Unraveling the role of angiogenesis in cancer ecosystems," *Frontiers in oncology*, vol. 8, 2018.

[57] M. R. Zaidi and G. Merlino, "The two faces of interferon-γ in cancer," *Clinical Cancer Research*, vol. 17, no. 19, pp. 6118–6124, 2011.

[58] Y. e. a. Wang, "Cpt1a-mediated fatty acid oxidation promotes colorectal cancer cell metastasis by inhibiting anoikis," *Oncogene*, vol. 37, pp. 6025–6040, 2018.

[59] A. e. a. Pakiet, "Changes in lipids composition and metabolism in colorectal cancer: a review.," *Lipids in health and disease*, vol. 18, 2019.

[60] J. Lin, Z. Zhu, H. Xiao, M. R. Wakefield, V. A. Ding, Q. Bai, and Y. Fang, "The role of il-7 in immunity and cancer," *Anticancer research*, vol. 37, no. 3, pp. 963–967, 2017.

[61] J. Gao, L. Zhao, Y. Y. Wan, and B. Zhu, "Mechanism of action of il-7 and its potential applications and limitations in cancer immunotherapy," *International Journal of Molecular Sciences*, vol. 16, no. 5, pp. 10267–10280, 2015.

[62] e. a. Deep Kwatra, "Methanolic extracts of bitter melon inhibit colon cancer stem cells by affecting energy homeostasis and autophagy," *Evidence-Based Complementary and Alternative Medicine*, vol. 2013, 2013.

[63] W. J. t. Broitman S.A., Cerda S., "Cholesterol metabolism and colon cancer.," *Prog Food Nutr Sci.*, vol. 17, pp. 1–40, 1993.

[64] S. e. a. Di Franco, "Role of type i and ii interferons in colorectal cancer and melanoma," *Frontiers in immunology*, vol. 8, 2017.

[65] J. Wang, X. Sun, W. Mao, W. Sun, J. Tang, M. Sui, Y. Shen, and Z. Gu, "Tumor redox heterogeneity-responsive prodrug nanocapsules for cancer chemotherapy," *Advanced Materials*, vol. 25, no. 27, pp. 3670–3676, 2013.

[66] B. S. Reddy and D. A. K. M. Shamsudden, "Nutritional factors and colon cancer," *Critical Reviews in Food Science and Nutrition*, vol. 35, no. 3, pp. 175–190, 1995. PMID: 7632353.

[67] S. e. a. Tugues, "New insights into il-12-mediated tumor suppression," *Cell death and differentiation*, vol. 22, pp. 237–246, 2015.

[68] P. G. Bradford and A. B. Awad, "Phytosterols as anticancer compounds," *Molecular Nutrition & Food Research*, vol. 51, no. 2, pp. 161–170, 2007.

[69] C. D. . Pino MS, "The chromosomal instability pathway in colon cancer," *Gastroenterology*, vol. 138, pp. 2059–2072, 2010.

[70] R. Stewart, S. Hammond, M. Oberst, and R. Wilkinson, "The role of fc gamma receptors in the activity of immunomodulatory antibodies for cancer," *Journal for ImmunoTherapy of Cancer*, vol. 2, 2014.

[71] R. . . . Radinsky, "Paracrine growth regulation of human colon carcinoma organ-specific metastasis," *Cancer Metast Rev*, vol. 12, 1993.

[72] B. Y. D. Y. S. . G. V. Sivaprakasam, S., "Short-chain fatty acid transporters: Role in colonic homeostasis.," *Comprehensive Physiology*, vol. 8, pp. 299–314, 2017.

[73] T. Wang, "Structural segregation of gut microbiota between colorectal cancer patients and healthy volunteers," *The Isme Journal*, vol. 6, p. 320–329, 2012.

[74] M. R. Wilson, Y. Jiang, P. W. Villalta, A. Stornetta, P. D. Boudreau, A. Carrá, C. A. Brennan, E. Chun, L. Ngo, L. D. Samson, B. P. Engelward, W. S. Garrett, S. Balbo, and E. P. Balskus, "The human gut bacterial genotoxin colibactin alkylates dna," *Science*, vol. 363, no. 6428, 2019.

[75] B. Caterson and J. Melrose, "Keratan sulfate, a complex glycosaminoglycan with unique functional capability," *Glycobiology*, vol. 28, pp. 182–206, 01 2018.

[76] e. a. Leiphrakpam, Premila D., "Role of keratan sulfate expression in human pancreatic cancer malignancy," *Scientific Reports*, vol. 9, p. 9665, 2019.

[77] C. Zhu, "Somatic mutation of dnah genes implicated higher chemotherapy response rate in gastric adenocarcinoma patients," *Journal of Translational Medicine*, vol. 17, 2019.

[78] C. D. House, C. J. Vaske, A. M. Schwartz, V. Obias, B. Frank, T. Luu, N. Sarvazyan, R. Irby, R. L. Strausberg, T. G. Hales, J. M. Stuart, and N. H. Lee, "Voltage-gated na+ channel scn5a is a key regulator of a gene transcriptional network that controls colon cancer invasion," *Cancer research*, vol. 70, p. 6957—6967, September 2010.

[79] G. M. e. a. Bourroul, "The destruction complex of beta-catenin in colorectal carcinoma and colonic adenoma," *Einstein (Sao Paulo, Brazil)*, vol. 14, pp. 135–142, 2016.

[80] T. e. a. Zhan, "Wnt signaling in cancer," *Oncogene*, vol. 36, pp. 1461–1473, 2017.

[81] F. I. Daniel, K. Cherubini, L. S. Yurgel, M. A. Z. de Figueiredo, and F. G. Salum, "The role of epigenetic transcription repression and dna methyltransferases in cancer," *Cancer*, vol. 117, no. 4, pp. 677–687, 2011.

[82] W. Lim, C. Park, M. K. Shim, Y. H. Lee, Y. M. Lee, and Y. Lee, "Glucocorticoids suppress hypoxia-induced cox-2 and hypoxia inducible factor-1α expression through the induction of glucocorticoid-induced leucine zipper," *British Journal of Pharmacology*, vol. 171, no. 3, pp. 735–745, 2014.

[83] S. M. N. e. a. Udden, "Nod2 suppresses colorectal tumorigenesis via downregulation of the tlr pathways," *Cell reports*, vol. 19, pp. 2756–2770, 2017.

[84] J. e. a. Peñarando, "A role for endothelial nitric oxide synthase in intestinal stem cell proliferation and mesenchymal colorectal cancer.," *BMC biology*, vol. 16, 2018.

[85] G. A. de Oliveira, R. Y. Cheng, L. A. Ridnour, D. Basudhar, V. Somasundaram, D. W. McVicar, H. P. Monteiro, and D. A. Wink, "Inducible nitric oxide synthase in the carcinogenesis of gastrointestinal cancers," *Antioxidants & Redox Signaling*, vol. 26, no. 18, pp. 1059–1077, 2017. PMID: 27494631.

[86] E. Voronov and R. N. Apte, "Il-1 in colon inflammation, colon carcinogenesis and invasiveness of colon cancer," *Cancer microenvironment : official journal of the International Cancer Microenvironment Society*, vol. 8, pp. 187–200, 2015.

[87] B. e. a. Rovcanin, "Mitotic crossover–an evolutionary rudiment which promotes carcinogenesis of colorectal carcinoma," *World journal of gastroenterology*, vol. 20, pp. 12522–12525, 2014.

[88] S. Song and Y. Yu, "Progression on citrullination of proteins in gastrointestinal cancers," *Frontiers in Oncology*, vol. 9, p. 15, 2019.

[89] B. e. a. Yang, "Small molecule rl71 targets serca2 at a novel site in the treatment of human colorectal cancer," *Oncotarget*, vol. 6, pp. 37613–37625, 2015.

[90] A. e. a. Crespo, "Heparan sulfate proteoglycans undergo differential expression alterations in left sided colorectal cancer, depending on their metastatic character," *BMC cancer*, vol. 18, 2018.

[91] R. e. a. Mizuno, "The role of tumor-associated neutrophils in colorectal cancer," *International journal of molecular sciences*, vol. 20, p. 529, 2019.

[92] T. Reya, M. O'Riordan, R. Okamura, E. Devaney, K. Willert, R. Nusse, and R. Grosschedl, "Wnt signaling regulates b lymphocyte proliferation through a lef-1 dependent mechanism," *Immunity*, vol. 13, no. 1, pp. 15 – 24, 2000.

[93] P. J. Morin, "β-catenin signaling and cancer," *BioEssays*, vol. 21, no. 12, pp. 1021–1030, 1999.

[94] T. Y. K. S. e. a. Ukegawa, JI., "Growth-promoting effect of muscarinic acetylcholine receptors in colon cancer cells," *Journal of Cancer Research and Clinical Oncology*, vol. 129, pp. 272–278, 2003.

[95] M. e. a. Moossavi, "Role of the nlrp3 inflammasome in cancer," *Molecular cancer*, vol. 17, 2018.

[96] V. e. a. Derangére, "Liver x receptor β activation induces pyroptosis of human and murine colon cancer cells," *Cell Death And Differentiation*, vol. 21, p. 1914–1924, 2014.

[97] D. Pennica, T. A. Swanson, J. W. Welsh, M. A. Roy, D. A. Lawrence, J. Lee, J. Brush, L. A. Taneyhill, B. Deuel, M. Lew, C. Watanabe, R. L. Cohen, M. F. Melhem, G. G. Finley, P. Quirke, A. D. Goddard, K. J. Hillan, A. L. Gurney, D. Botstein, and A. J. Levine, "Wisp genes are members of the connective tissue growth factor family that are up-regulated in wnt-1-transformed cells and aberrantly expressed in human colon tumors," *Proceedings of the National Academy of Sciences*, vol. 95, no. 25, pp. 14717–14722, 1998.

[98] A.-S. e. a. Bélanger, "Regulation of ugt1a1 and hnf1 transcription factor gene expression by dna methylation in colon cancer cells," *BMC Molecular Biology*, vol. 11, 2010.

[99] L. D. P. J. e. a. . Dolcet, X., "Nf-kb in development and progression of human cancer," *Virchows Arch*, vol. 446, pp. 475–482, 2005.

[100] V. Huber, S. Fais, M. Iero, L. Lugini, P. Canese, P. Squarcina, A. Zaccheddu, M. Colone, G. Arancia, M. Gentile, E. Seregni, R. Valenti, G. Ballabio, F. Belli, E. Leo, G. Parmiani, and L. Rivoltini, "Human colorectal cancer cells induce t-cell death through release of proapoptotic microvesicles: Role in immune escape," *Gastroenterology*, vol. 128, no. 7, pp. 1796 – 1804, 2005.
